# Single-bubble water boiling on small heater under Earth’s and low gravity

**DOI:** 10.1038/s41526-018-0055-y

**Published:** 2018-11-02

**Authors:** Ezinwa Elele, Yueyang Shen, John Tang, Qian Lei, Boris Khusid

**Affiliations:** Otto H. York Department of Chemical, Biological & Pharmaceutical Engineering, New Jersey Institute of Technology, Newark, NJ USA

## Abstract

Today’s trends for enhancing boiling heat transfer in terrestrial and space applications focus on removal of bubbles to prevent formation of a vapor layer over the surface at high overheat. In contrast, this paper presents a new boiling regime that employs a vapor–air bubble residing on a small heater for minutes and driving cold water over the surface to provide high heat flux. Single-bubble boiling of water was investigated under normal gravity and low gravity in parabolic flights. Experiments demonstrated a negligible effect of gravity level on the rate of heat transfer from the heater. Due to self-adjustment of the bubble size, the heat flux provided by boiling rose linearly up with increasing heater temperature and was not affected by a gradually rising water temperature. The fast response and stable operation of single-bubble boiling over a broad range of temperatures pave the way for development of new devices to control heat transfer by forming surface domains with distinct thermal properties and wettability. The bubble lifetime can be adjusted by changing the water temperature. The ability of heating water on millimeter scales far above 100 °C without an autoclave or a powerful laser provides a new approach for processing of biomaterials and chemical reactions.

## Fluids: A quicker way to boil water

A mechanism by which light-weight heaters can more effectively boil water is identified by researchers in the USA. Increasing the rate at which a fluid is heated is hampered by the formation of a layer of vapor on the surface of a heater, which prevents heat flow. This is particularly problematic for small heaters such as those required in space applications. Boris Khusid and his colleagues from the New Jersey Institute of Technology now demonstrate a new boiling regime that takes advantage of an air-vapor bubble that forms on a smaller heater. This bubble drives cold water to the surface and improves the flow of heat. This effect was investigated both under normal gravity and in a low-gravity environment created by parabolic flights, with gravity exhibiting only a negligible influence on the rate of heat transfer.

## Introduction

Miniaturization of electronic and photonic systems is challenged by a dramatic increase in the power dissipation per unit volume with the occurrence of hot spots where the heat flux is much higher than the average. Force-flow cooling by gas or liquid appears insufficient to remove local high heat fluxes.^1,2^ Boiling that involves evaporation of liquid in a hot spot and condensation of vapor in a cold region can remove a significantly larger amount of heat through the latent heat of vaporization.^3^ It is therefore considered as the most promising cooling technology for future microgravity applications.^4,5^ Boiling begins at a certain value of the heater temperature, termed the onset of nucleate boiling, with the appearance of bubbles in surface imperfections. The classical scenario of the pool boiling on Earth under normal gravity, g_E_, in which a heater is submerged in a stagnant liquid is that a bubble, once formed, will grow driven by liquid evaporation and detach from the surface.^3^ As the bubble departs, a hot liquid is pushed away from the surface and replaced with a cooler liquid from the bulk. An increase in the heater temperature, *T*_h_, activates more nucleation sites and accelerates the vapor production, bubble growth and departure. The nonlinear nature of these phenomena causes the heat flux *q*_h_ to rise rapidly with the heater superheat^6,7^ as *q*_h_~(*T*_h_−*T*_sat_)^m^, where *T*_sat_ is the liquid saturation temperature at a given pressure and m ranges from 3 to 5. Eventually the heat flux reaches a maximum (termed the critical heat flux) when bubbles growing in adjacent cites merge together and cover the heater surface with vapor. Heat transfer is then drastically reduced as the heated surface is totally blanketed with an insulating vapor film.^3^

Extensive studies of nucleate boiling on small heaters were conducted over the past two decades under normal gravity, low gravity and hypergravity.^8–18^ It was demonstrated that the relative contribution of the buoyancy and surface tension to the energy transfer away from the heater can be characterized by the ratio of the characteristic heater size *L*_h_ to the capillary length ${\mathrm{L}}_{\mathrm{c}} = \sqrt {{\mathrm{\gamma }}_{\mathrm{l}}/{\mathrm{g}}\left( {{\mathrm{\rho }}_{\mathrm{l}}{\mathrm{ - \rho }}_{\mathrm{v}}} \right)}$ specified by the liquid surface tension γ_1_, liquid ρ_1_ and vapor ρ_v_ densities, and gravity acceleration g. The classical scenario of boiling occurs at a sufficiently large *L*_h_/*L*_c_ (large heaters or high gravity) when the buoyancy facilitates removal of bubbles from the surface. A threshold depends on the heater geometry and liquid properties. Boiling at small *L*_h_/*L*_c_ (small heaters or low gravity) is dominated by capillary forces and thermocapillary convection around bubbles.^15–18^ Nucleated bubbles in this case are observed to slide over the heater, detach (even in microgravity due to the vapor recoil) and hover close to the heater, coalescing with satellite bubbles.^8–18^ Once bubbles form a vapor layer covering the heated surface, heat transfer is dramatically reduced.

The appearance of a vapor film on the surface at high superheat is the bottleneck to the performance of boiling heat transfer on relatively large and small heaters. Accordingly, current methods for heat transfer enhancement focus on removal of bubbles to prevent the creation of a vapor film.^3–5,10–12,18^ In contrast, we present a new boiling regime of water in which a vapor-air bubble anchored to a small heater for minutes at temperature up to about 280 °C provides a heat flux up to ~ 1.2 MW/m^2^, even though this value lies within the range of maximum heat fluxes on horizontal surfaces under normal gravity.^3^

## Results

**Fig. 1:**
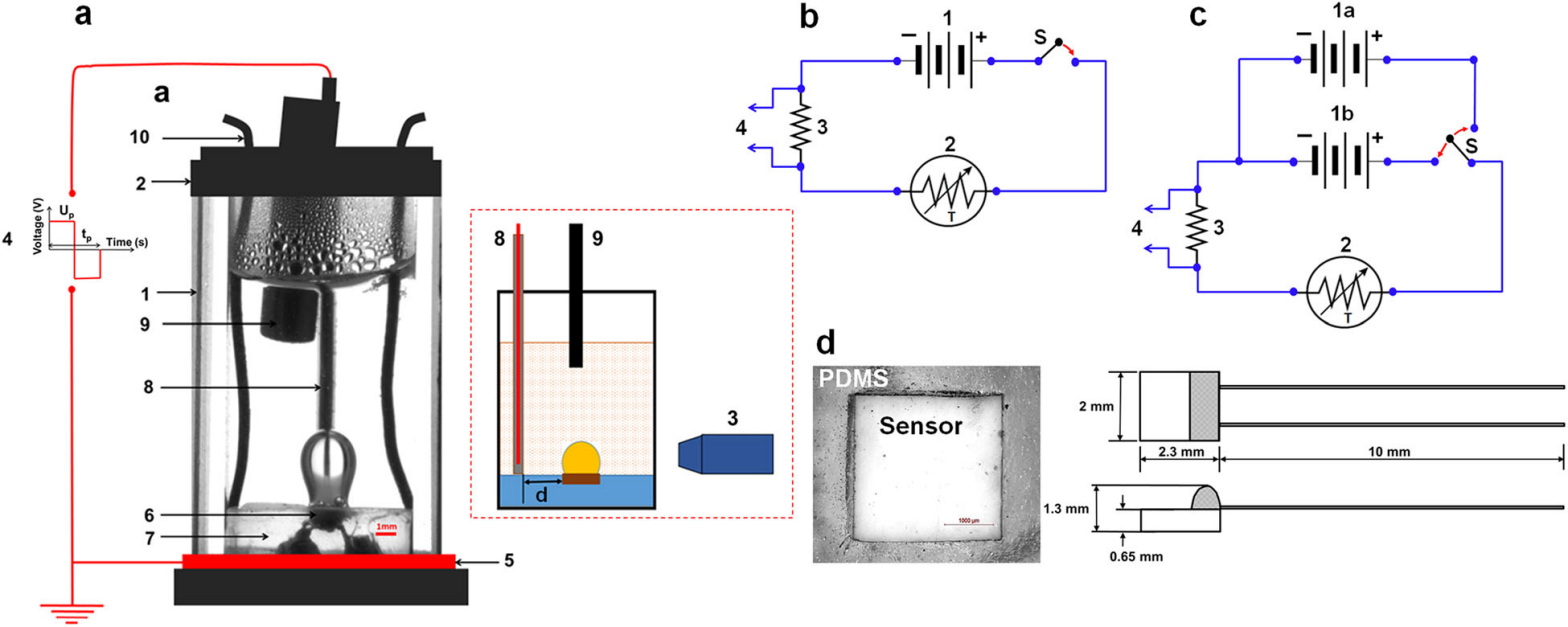
Cell: **a** Front and side views: 1, cuvette with liquid; 2, cover; 3, camera, signal recorded by a laptop over the entire experiment; 4, connection to amplifier for generating high voltage pulses of alternating polarity; 5, grounded electrode; 6, temperature resistance sensor (heater); 7, PDMS slab; 8, energized electrode coated with Teflon at 2 mm from the heater, 9, temperature probe in ground experiments; 10, connection to heating circuit. Electrical circuits: **b** flight: 1, heating DC power source; **c** Earth: 1a, heating DC power source; 1b, 3 V DC power source; S, switch; (both **b**, **c**) 2, heater; 3, resistor; 4, connection to acquisition system; **d** photo of platinum sensor P0K1.232.4 W.B.010 in PDMS slab whose silver wires (diameter 0.25 mm, length 10.0 mm) were soldered to power lead copper wires (Gauge 36 copper wire, length ~4 mm); sensor sketch reproduced with permission from Innovative Sensor Technology, Las Vegas, NV

**Fig. 2:**
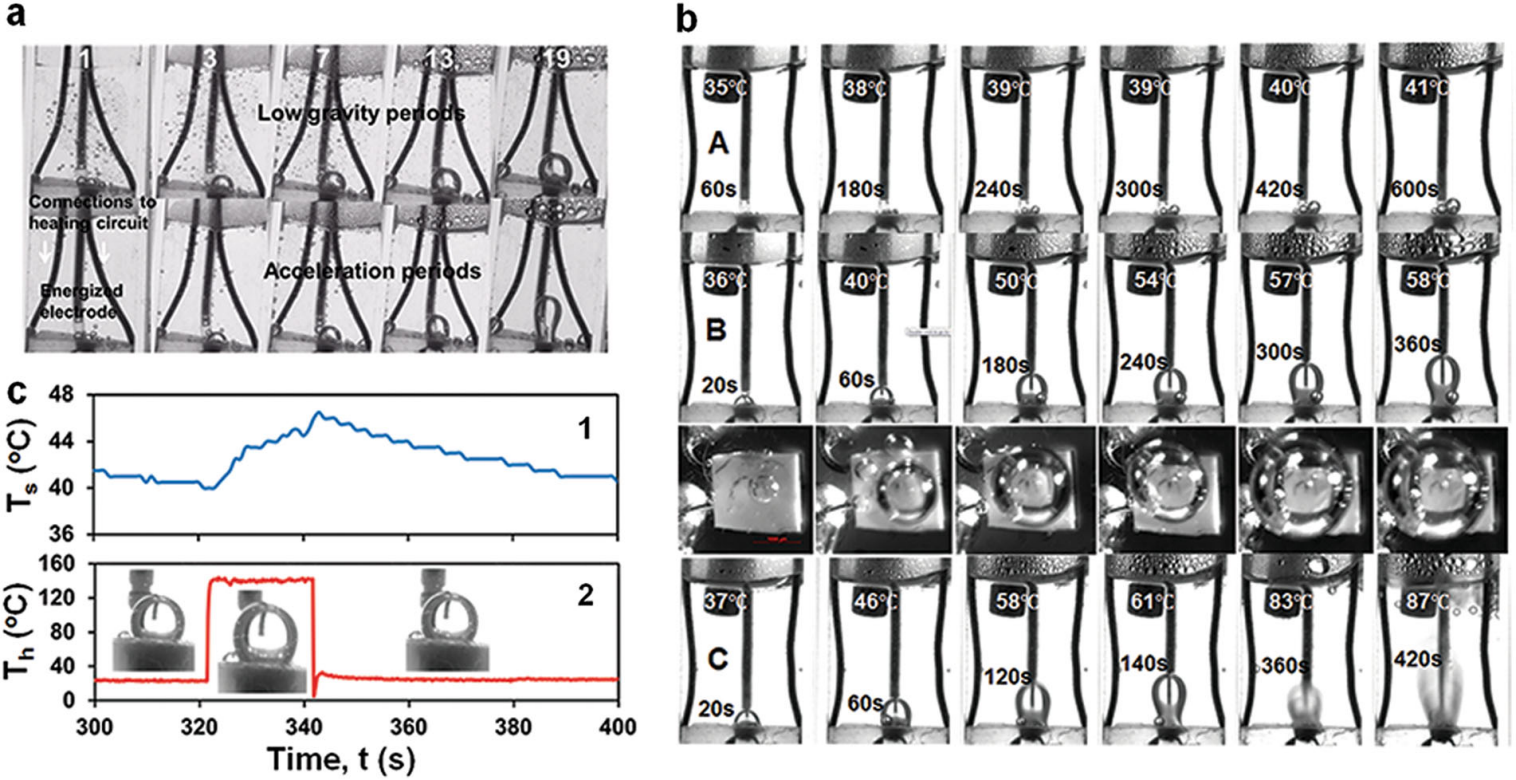
Thermal regimes: **a** Flight, parabolas (number shown); 22.4 V DC & 4 kV/20 Hz pulses applied in freefall (top row) and switched off during acceleration (bottom row); **b** Earth, continuous heating, no HV pulses; T_s_ and heating time shown; applied V DC: 10 (**A**); 20 (**B**); 30 (**C**) see heat flux for **A**, **B**, **C** in Fig. 4a. **c** Earth, heating cycles 20 V DC & 4 kV/20 Hz pulses 20 s on/60 s off, temperatures: 1, T_s_; 2, T_h_

**Fig. 3:**
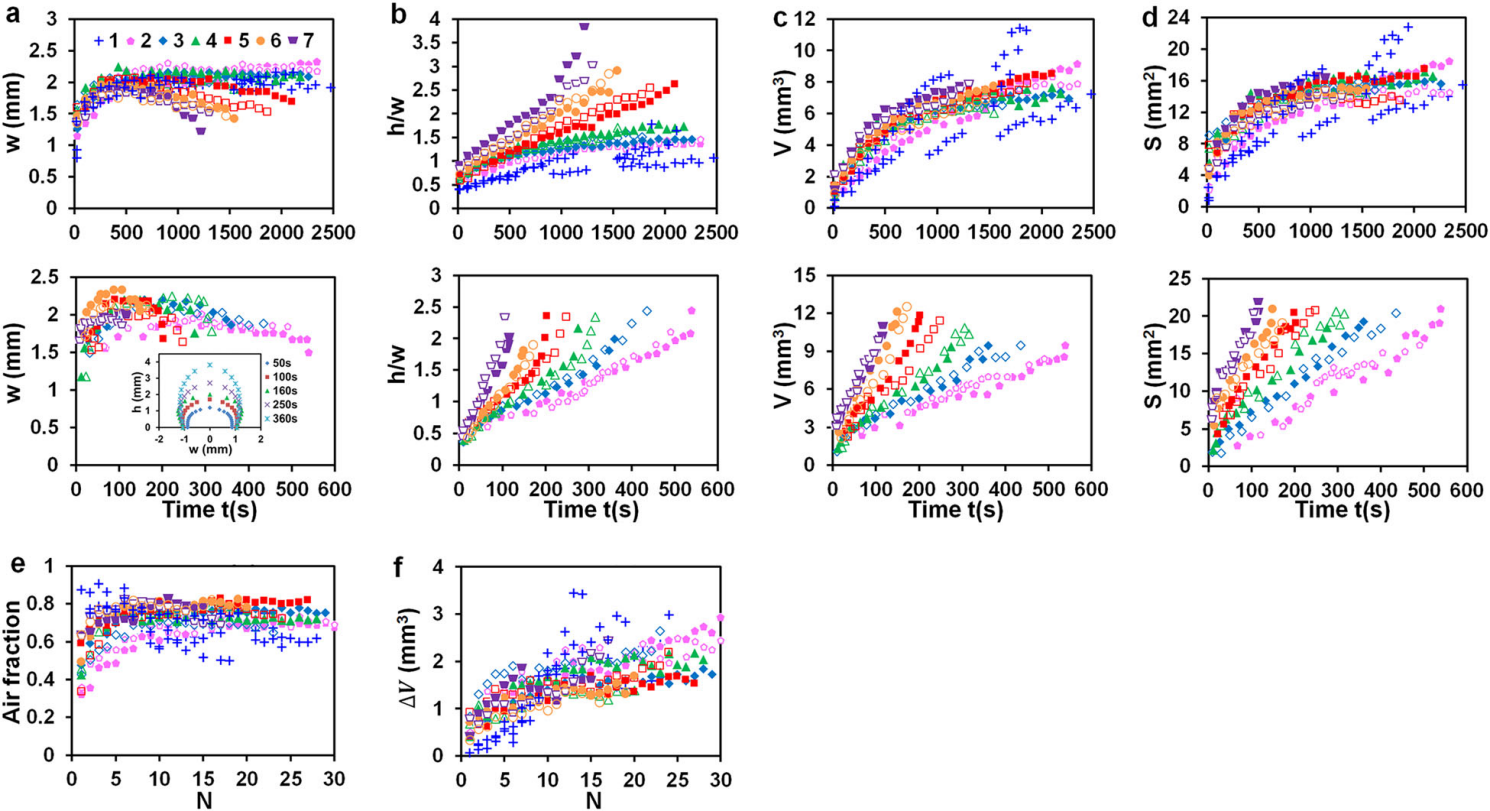
Thermal regimes. **a**–**d** Bubble width w, height h, ratio h/w, volume V, and cap surface area S in heating period; curves terminate after departure of the first bubble: Flight 1, 20 V DC & 3 kV/20 Hz; 22.4 V DC & 4 kV/20 Hz; 22.4 V DC & 4 kV/10 Hz, pulses applied in freefall and switched off during acceleration; Earth 2–7, heating cycles 20 s on/60 s off (top row) and continuous heating (bottom row) with 4 kV/20 Hz pulses (empty symbols) and without HV pulses (filled symbols); applied V DC: 15 (2); 20 (3); 22.4 (4); 25 (5); 30 (6); 35 (7); inset 20 V DC. **e**, **f** Changes of the bubble volume Δ*V* due to water vapor condensation as heating was turned off and the air fraction in the bubble (1−ΔV/V) vs. the number N of heating cycles for regimes listed in (**a**–**d**). V, S, and ΔV were computed by integration of the shape profile along the bubble image (SI1). Results of statistical analysis of measurements are listed in SI1

**Fig. 4:**
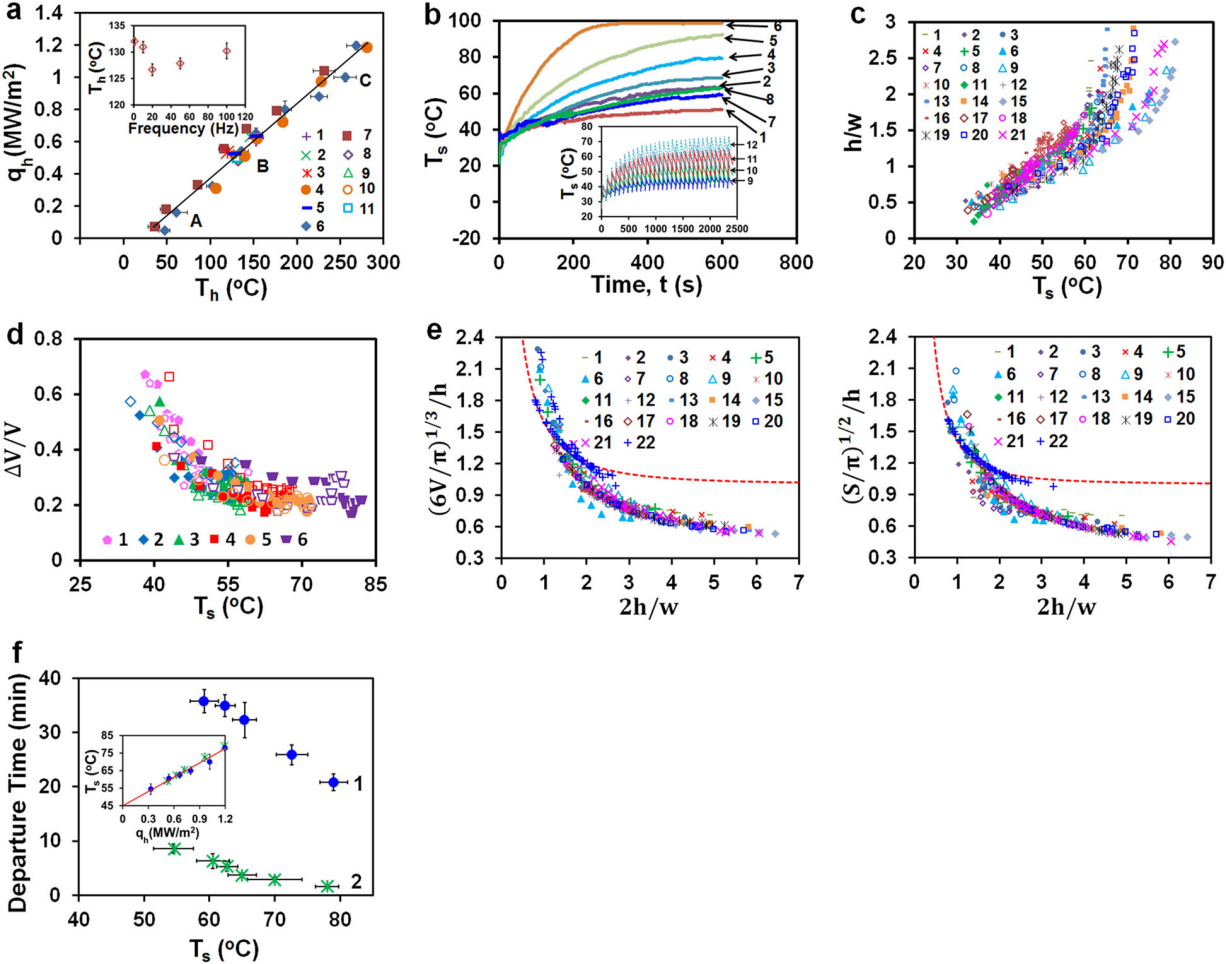
Effects of increasing heating DC voltage: **a** Stabilized heat flux q_h_ vs. heater temperature T_h_. Flight: 1, 20 V DC & 3 kV/20 Hz; 2, 22.4 V DC & 4 kV/20 Hz; 3, 22.4 V DC & 4 kV/10 Hz; Earth, heating cycles 20 s on/60 s off: 4, no HV pulses (points for 15, 20, 22.4, 25, 30, 35 V DC); 5, with 4 kV/20 Hz pulses (points for 20, 22.4 V DC); Earth, continuous heating: 6, no HV pulses (points for 5, 10, 15, 20, 22.4, 25, 27.5, 30, 35 V DC); 7, with 4 kV/20 Hz pulses (points for 5, 10, 15, 20, 22.4, 25, 30 V DC); 8–11, 20 V DC with 4 kV pulses at 1 (8); 10 (9); 50 (10); 100 (11) Hz. Points **A**, **B**, **C** mark regimes in Fig. 2b. Inset: Earth, continuous heating 20 V DC: T_h_ vs. frequency of 4 kV pulses. **b** Earth, T_s_, continuous heating: without HV pulses for 15 (1), 20 (2), 22.4 (3), 25 (4), 30 (5), 35 (6) V DC and with 4 kV/20 Hz pulses for 20 (7), 22.4 (8) V DC. Inset: heating cycles 20 s on/60 s off without HV pulses for: 15 (9), 20 (10), 25 (11), 30 (12) V DC. **c** Earth, bubble height to width ratio h/w vs. T_s_ for continuous heating: no HV pulses for 15 (1), 20 (2); 22.4 (3), 25 (4); 30 (5); 35 (6) V DC and with 4 kV/20 Hz pulses for 20 (7), 22.4 (8), 35 (9) V DC; heating cycles 20 s on/60 s off: no HV pulses for 15 (10), 20 (11), 22.4 (12), 25 (13), 30 (14), 35 (15) V DC and with 4 kV/20 Hz pulses for 15 (16), 20 (17), 22.4 (18), 25 (19), 30 (20), 35 (21) V DC. **d** Earth, relative changes of bubble volume Δ*V*/*V* as heating turned off vs. T_s_ for heating cycles 20 s on/60 s off with 4 kV/20 Hz pulses (empty symbols) and without HV pulses (filled symbols): 15 (1), 20 (2), 22.4 (3), 25 (4), 30 (5), 35 (6) V DC. (**e**) The bubble volume V and cap surface area S normalized by the bubble height h vs. bubble height to width ratio 2 h/w for experiments on Earth, 1–21 as listed in (**c**), and in flight 22: 20 V DC & 3 kV/20 Hz; 22.4 V DC & 4 kV/20 Hz; 22.4 V DC & 4 kV/10 Hz. The dashed line represents the spherical cap. (**f**) Earth, times of bubble departure vs. liquid temperature T_s_ for 1, heating cycles 20 s on/60 s off and 2, continuous heating. Points for 15 V DC (only for regime 2 as a bubble remained on the heater after 40 min of cycles), 20, 22.4, 25, 30, 35 V DC (data with and without 4 kV/20 Hz pulses within error bars) arranged from left to right. Inset: T_s_ vs. heat flux. Results of statistical analysis of measurements are listed in SI1. Error bars in (**a**) and (**f**) represent standard deviations

**Fig. 5:**
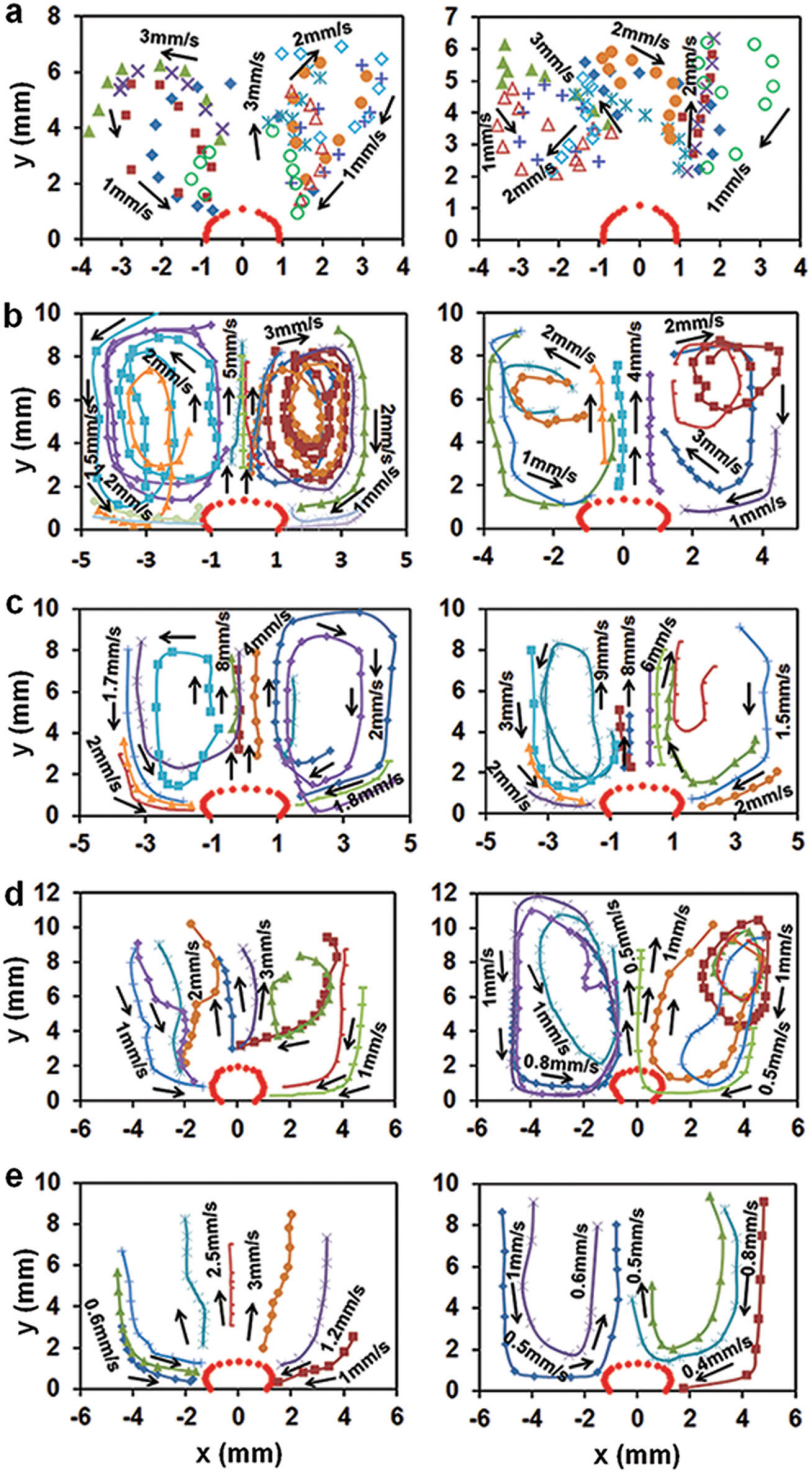
Flow patterns in the cuvette vertical plane: **a** Flight, left: freefall, 22.4 V DC with 4 kV/20 Hz pulses; *right*: acceleration, no heating and pulses. **b**, **c** Earth, continuous heating: **b** 22.4 DC, **c** 30 V DC; with 4 kV/20 Hz pulses (left), without pulses (right). **d**, **e** Earth, heating cycles 20 V DC 20 s on/60 s off: **d** with 4 kV/20 Hz pulses; **e** without HV pulses; heating ON (left), OFF (right). Symbols indicate trajectories of 10 individual microbubbles for **a** and individual 75–90 µm blue polyethylene microspheres (1.00 g/cm^3^, Cospheric, Santa Barbara, CA) on Earth: **b** 12 particles for left and 11 for right; **c** 12 for left and right; **d** 10 for left and right; **e** 8 for left and 5 for right

**Fig. 6:**
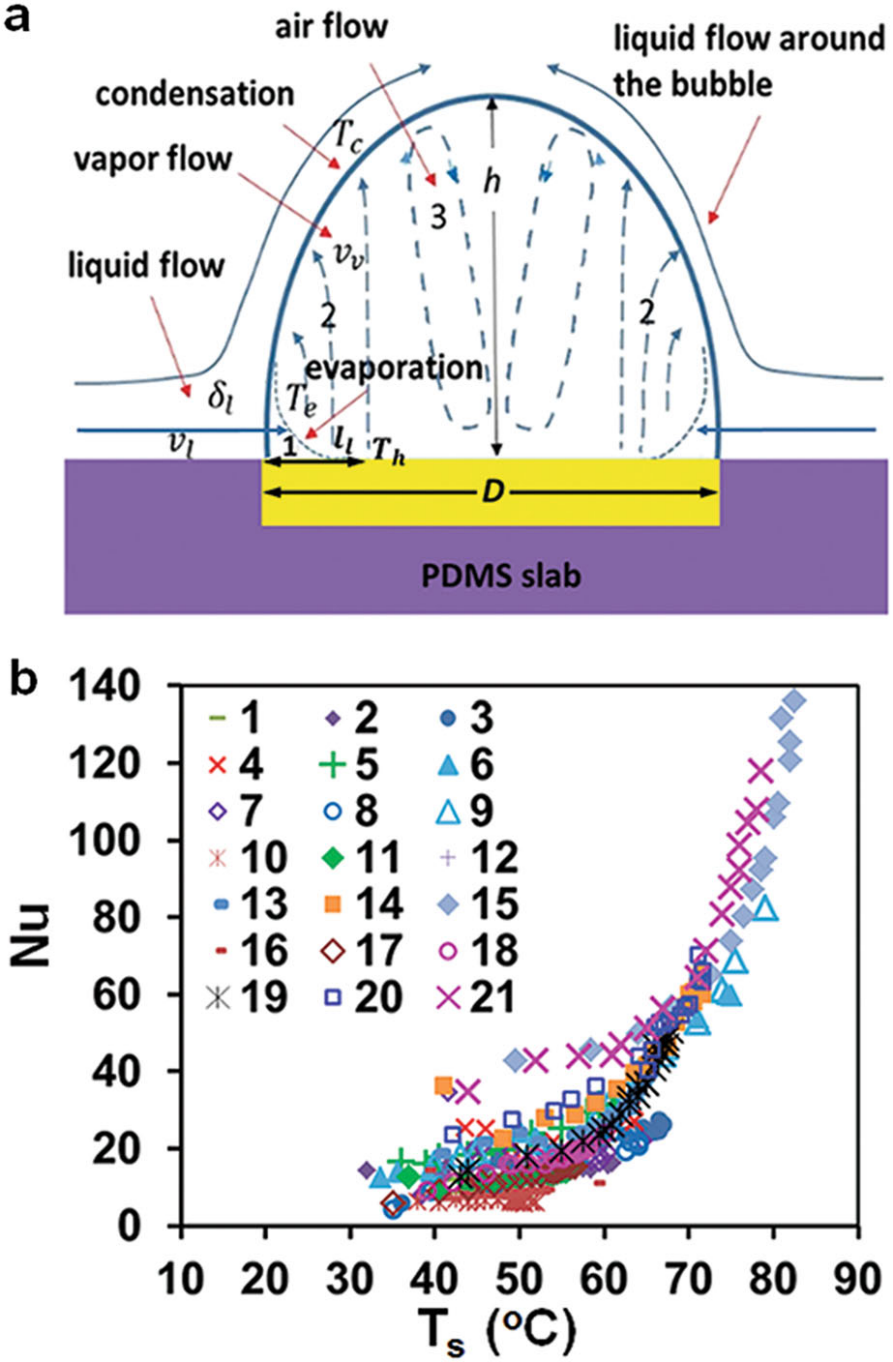
**a** Schematic of single-bubble boiling: 1, layer of thickness δ_1_ and length l_1_ at the bubble footprint where cold liquid flowing into the bubble with velocity *v*_1_ vaporizes; 2, vapor streaming toward the bubble cap with velocity v_v_; 3, non-condensable air constituents accumulating away from the bubble cap; *T*_e_ and *T*_c_, evaporation and condensation temperatures; *h* and *D*, bubble height and base diameter. **b** Earth, the Nusselt number Nu for heat transfer between bubble cap and surrounding liquid vs. *T*_s_ for continuous heating and heating cycles, 1–21 as listed in Fig. 4c

The conditions of experiments carried out in parabolic flights and on Earth are listed in (SI1). Low gravity measurements were conducted in 3 days of parabolic flights in NASA Boeing 727. A flight provided two sets of consecutive parabolic arcs, each with 15-s freefall at acceleration ~ 10^−2^g_E_ preceded and followed by periods of acceleration ~ 1.3g_E_ for 50–60 s where g_E_ is the normal gravity acceleration; the second set also included two parabolas simulating the gravity of the Moon (1.62 m/s^2^) and Mars (3.71 m/s^2^). Following guidelines,^19^ a flight setup was designed to withstand “crash g-forces” up to 9g_E_ along horizontal axis, up to 2g_E_ along positive vertical axis and up to 6g_E_ along negative vertical axis (Supplementary information, SI1). The setup was equipped with two cuvettes shown in Fig. 1 for simultaneous testing of different liquids. Experiments were carried out on 3 M Novec HFE-7100 (3 M, St. Paul, MN) and distilled water from a local pharmacy with conductivity σ_l_ ~ 2·10^−4^ S/m and dielectric constant ε_1_~ 78 measured before experiments. Here we present data only for water. Due to the limited space of the paper, experiments on HFE-7100 for which a conventional boiling regime was observed will be reported elsewhere. For water at room temperature, *L*_c_ equals 2.7 mm on Earth and 27 mm for freefall. A cuvette (Fig. 1) was loaded with 1 mL of water and then closed with a plastic lid. As a liquid in a cooling system utilized over a long period of time usually accumulates dissolved air, experiments were carried out with the cuvette lid that was not airtight to maintain atmospheric pressure inside the cuvette. A platinum temperature resistance sensor, serving as a heater, was embedded into a polydimethylsiloxane (PDMS) slab such that the heating surface was in contact with water (Fig. 1d). The ratios of the heating surface width and length (Fig. 1d) to the capillary length were respectively 0.74, 0.85 for experiments on Earth and 0.074, 0.085 in flight. The interior contact angles of water on the heater surface and PDMS measured at room temperature were respectively 74 ± 3° (hydrophilic) and 107 ± 2° (hydrophobic). The heater was connected in series with a resister R_0_ and a direct current (DC) power source that provided voltage U_DC_ to the heater (Fig. 1). The voltage drop across this resistor U_R_ was measured to compute the voltage drop on the heater ΔU_h_ = U_DC_−U_R_, the electrical current *I*_h_ = *U*_R_/*R*_0_, the power *I*_h_Δ*U*_h_ that varied from about 0.5 W to 5.5 W and the heater resistance *R*_h_ = Δ*U*_h_/*I*_h_ that was used for calculating its temperature T_h_ from the linear calibration curve *R*_h_ vs. *T*_h_. The values of heat flux *q*_h_ = *I*_h_Δ*U*_h_/*S*_h_ reported in Figs. 2–6 correspond to the total power provided to the heater. Expressions^20^ were used to estimate the heat loss from the heater through the power lead wires and directly into the PDMS slab. Calculations presented in SI1 indicate that the heat loss increased with raising the heater temperature from about 9% at *T*_h_ ≈ 50 °C to 13% at *T*_h_ ≈ 270 °C. Additional two parameters were measured on Earth due to flight limitations: (i) the temperature T_s_ slightly below the water surface by probe 9 in Fig. 1a to estimate the bulk liquid temperature and (ii) the heater temperature *T*_h_ when the heating power source was turned off by applying 3 V DC to the heater (Fig. 1c). For the latter, the generated power ~ 0.075 W might raise the heater temperature by ~ 2°C.

Since electric fields are widely used to enhance boiling heat transfer,^18,21–24^ the cuvette was equipped with electrodes to investigate the field effects on single-bubble boiling. Conventional electric techniques are limited to low conducting liquids because of using bare electrodes inserted into the liquid. To avoid this limitation, a train of successive rectangular high-voltage (HV) pulses of alternating polarity, *U*_p_ = 3–4 kV at frequency 1/t_p_ up to 100 Hz, was applied to water via the insulated energized electrode inserted into the water and the grounded electrode placed under the cuvette (Fig. 1a). The power supplied by HV pulses was less than 0.2 W (SI1). As the electric stress exerted on a liquid is proportional to the square of the field strength, the application of these pulses kept the electric stress at a constant level. The motion of charge carriers in a liquid subjected to an electric field depends on the ratio^25^ between the charge relaxation time *t*_rel_ = ε_0_ε_1_/σ_1_ and the period of HV pulses *t*_p_, where ε_0_ is the vacuum permittivity. As *t*_rel_~ 5μs≪*t*_p_ in our experiments, ions in water followed the field, thereby reducing the accumulation of charge due to voltage reversals. The proposed design offers the ability of applying an electric force to liquids with much higher electrical conductivity as the chance of short circuit, sparking, and electro-corrosion are drastically reduced.

All flight heating tests were performed in the presence of HV pulses (SI1). Heating DC voltage 20 V or 22.4 V and HV pulses were simultaneously turned on when the aircraft began to freefall. Once the aircraft began to accelerate, they were turned off to avoid the contribution of buoyancy and electric field driven convection. They were also turned off as the aircraft maneuvered for about 10 min to begin flying the second set of parabolas.

A large bubble rapidly formed on the heater during the first freefall. Its footprint was gradually increasing in size, until anchoring on the heater edges after a couple of minutes. It was staying on the heater over the first set of parabolas and detached during the acceleration period of one of parabolas during the second set (Fig. 2a, SI2, video1). Specifically, a bubble formed in the first freefall was remaining on the heater for total of 20 parabolas (32 min) in the first flight, 27 (41 min) in the second flight and 19 parabolas (36 min) in the third flight. As the firstly formed bubble detached, another one formed and stayed on the heater until the end of the flight. During a freefall, a bubble staying on the heater emitted sporadically tiny bubbles that were carried away with the flow. As the aircraft was accelerating, these tiny bubbles rose to the water surface and popped up due to buoyancy. The heater temperature and heat flux during a freefall period stabilized within 2 s after applying DC voltage. Variations of heat flux values from parabola to parabola measured at the same heating regime were lying within 2–4% (SI1).

Experiments on Earth were carried out under conditions of continuous heating and heating cycles with DC voltage 20 s on/60 s off (SI1). Variations of heat flux values measured at the same heating regime were lying within 1–6% (SI1). A consistent performance of single-bubble boiling was observed for both heating modes. To avoid rapid deterioration of the PDMS slab around the heater, most experiments were conducted for heater temperatures below 270 °C. Photos in Fig. 2b illustrate bubbles formed under continuous heating. Tiny bubbles appeared on the heater at 10 V DC for which T_h_ was below 100 °C (Fig. 2b, regime A) were mainly due to the release of air dissolved in water as its solubility decreased with temperature. A single bubble formed on the heater when T_h_ was above 100 °C. Side and top images in Fig. 2b B show the evolution of the first bubble appeared at 20 V DC. The first bubble formed at 30 V DC is shown on images in Fig. 2b regime C from 20 to 140 s. As can be seen in Fig. 2b regimes B & C, the bubble foot gradually expanded until reaching the heater edge. While the heater temperature T_h_ and heat flux q_h_ stabilized within several seconds after applying DC voltage, the bulk temperature of water for both heating modes gradually rose due to the low rate of heat transfer out of the cuvette. This feature was used to investigate the effect of water temperature on single-bubble boiling. The timescale of changes in water temperature T_s_ for continuous heating was about 5 min for voltage greater than 10 V DC (SI1). The amplitude of water temperature variation in a heating cycle was in the range 2–6 °C, gradually increasing with applied DC voltage. The timescale of changes in water temperature from cycle to cycle were much smaller than that for continuous heating since the electric power averaged over a cycle was four times smaller. Plots and photos in Fig. 2c illustrate variations of *T*_h_, *T*_s_ and the bubble size in a typical heating cycle. When the heater temperature dropped down once the heating was off, the bubble shrank due to condensation of water vapor (Fig. 2a in flight and Fig. 2c on Earth). While the heater temperature and heat flux did not change in the process of boiling, the height h, volume *V*, and cap surface area *S* of the pinned bubble were gradually increasing due to the rising water temperature (Fig. 3a–d). Fig. 3e, f illustrates a change in the bubble volume Δ*V* caused by vapor condensation after the heating was turned off and the air volume fraction in the bubble (calculated as 1−Δ*V*/*V*) with the number *N* of heating cycles.

Points A, B, C in Fig. 4a mark regimes shown in Fig. 2b. The maximum efficiency of HV pulses was achieved at 20 Hz (Fig. 4a inset) at which the heat flux increased by about 10% at the same heater temperature. In flight and in both heating modes on Earth, the heat flux *q*_h_ of a pinned bubble was found to rise linearly to 1.2 MW/m^2^ with increasing heater temperature *T*_h_ to about 280 °C as (Fig. 4a, SI1)

1 $${{q}}_{\mathrm{h}}\left( {{\mathrm{kW/m}}^2} \right) = \left( {4.63 \pm 0.15} \right)\left( {{T}_{\mathrm{h}} - 19.28^\circ {\mathrm{C}}} \right)$$

with the coefficient of determination *r*^2^ = 0.970. Deviations between values given by this equation and measurements are normally distributed random quantities at the 95% confidence level (SI1). The remarkable independence of *q*_h_ and *T*_h_ from a gradually rising water temperature (Fig. 4(b) was caused by self-adjustment of the bubble size. As Fig. 4(c, d) show, data points in Fig. 3 on the bubble height to width ratio h/w and the fraction of water vapor in the bubble ΔV/V being plotted against T_s_ fell within a relatively narrow band for all heating regimes up to T_s_~ 80°C. While the bubble size increased with T_s_, the fraction of water vapor in the bubble tended to decrease. Fig. 4(e) illustrates the dependence of the bubble volume and cap surface area normalized by bubble height to width ratio 2 h/w ratio; the bubble base diameter was about 2 mm in flight and on Earth. Data points for flight experiments fell close to the curve for a spherical cap and data for all experiments on Earth grouped together below this curve. A bubble residing on the heater eventually divided into two parts by forming a large bubble that departed from the heater and a small bubble pinned to the heater. The remaining bubble grew to about the same size and then divided by forming another departing bubble. This process repeated itself several dozens of times, each time faster and faster, and finally produced a vapor plume whose size was increasing with water temperature (Fig. 2(b) C, images at 360 s and 420 s, SI3, video2). The lifetime of the first bubble formed on the heater after applying DC voltage was much longer for heating cycles than for continuous heating due to a slowly rising water temperature (Fig. 4(f). For both heating modes, the departure of this bubble occurred in the range of liquid bulk temperatures T_s_~ 50−80°C and showed the same dependence on the heat flux (Fig. 4f inset, SI1):

2 $${{T}}_{\mathrm{s}}\left( {\,^\circ {\mathrm{C}}} \right) = \left( {27.2 \pm 0.93} \right){{q}}_{\mathrm{h}}\left( {{\mathrm{MW/m}}^2} \right) + 44.96 \pm 1.13^\circ {\mathrm{C}}$$

with the coefficient of determination *r*^2^ = 0.966. Deviations between values given by this equation and measurements are normally distributed random quantities at the 95% confidence level (SI1).

Flow velocities around a pinned bubble shown in Fig. 5 were computed by tracking individual tiny bubbles formed in flight (seen in Fig. 2a) and beads seeded in the water in Earth experiments. Two toroidal eddies circulating around the bubble in the opposite directions were formed in flight (Fig. 5a). They pushed the hot water away from the bubble interface into the bulk and the cooler water from the bulk toward the bubble base with velocity ~ 1–3 mm/s. Similar eddies and a narrow vertical plume, rising from the bubble top with the velocity nearly twice greater than in the vortex flows, appeared under heating on Earth (Fig. 5b–e). However, the plume contribution to the heat flux was remarkably insignificant (Fig. 4(a). The vortex flows intensity slightly increased with increasing the applied DC voltage and decreased as the heating was turned off. Application of HV pulses made the vortex flows more stable. In flight and on Earth, the top of a pinned bubble was observed to oscillate (SI3, video2) at frequencies v_b_~ 2−4Hz with amplitude A_b_ that increased with water temperature from 10–30 µm at T_s_~ 25 °C to 100 µm at T_s_~ 80°C and was not affected by HV pulses. Velocities of bubble oscillations ~v_b_A_b_ = (20−120)μm/s were much smaller than vortex flow velocities (Fig. 5).

The basic feature of a single-bubble boiling recorded in our experiments is the appearance of a large bubble that is staying for minutes on a relatively small heater on Earth (L_h_/L_c_ ~ 0.8) and in flight (L_h_/L_c_ ~ 0.08) up to 280 °C at the water bulk temperature up to 80 °C (Fig. 4). This bubble behavior was not observed in previous studies of water boiling on relatively large and small heaters under normal and low gravity. Specifically, bubbles of a similar size were reported at high heat fluxes and low subcooling in boiling water on large horizontal flat heaters on Earth (L_h_/L_c_≥1).^26–30^ They formed via vigorous lateral coalescence among small bubbles and departed from the heater in less than ~ 0.1–0.3 s. In parabolic flights, bubble liftoff diameters from about 2 mm to 20 mm were observed in boiling water on flat heaters for L_h_/L_c_~ 1.8^31^ and ~ 3.7.^32^ However, these bubbles formed, grew and departed in about 10–12 s. Depending on the level of subcooling, several different modes of boiling water on thin horizontal wires were observed in experiments on Earth^33–41^ for L_h_/L_c_~ 0.0046−0.370, and in drop towers and parabolic flights^42–44^ for L_h_/L_c_~ 0.0009−0.0030, where L_h_ is the wire radius: large bubble boiling, coexisting boiling, and explosive small bubble boiling. In particular, raising the heater temperature for saturated boiling under normal^33^ and low gravity^42^ gradually increased the heat flux and the size of a vapor bubble covering the wire until it became totally blanked. As a result, the local maxima and minima in the curve heat flux vs. heater temperature for saturated boiling on Earth were found to vanish at^33^ L_h_/L_c_≤0.0096. However, the formation of a large single bubble engulfing the wire was not observed in the Space Shuttle experiments^45^ on subcooled boiling at L_h_/L_c_~ 0.0003. But instead, a set of discreet bubbles formed on a wire, grew up to 4–6 mm and departed within about 10 min. An abrupt transition from the formation of individual bubbles which sprang from the heating surface in all radial directions to the formation of a thick, stable vapor film over the entire cylindrical heater was recorded for saturated boiling in parabolic flight^46^ at L_h_/L_c_~ 0.17. The lateral motion of bubbles over the heating surface leading to their coalescence were observed in subcooled boiling on single and twisted submillimeter wires on Earth^35–41^ and in parabolic flights.^43,44^ Bubbles with diameters ranging from about 0.01 to 1.6 mm and departing from the heater in ~0.1 to 0.3 s on Earth and ~1–18 s in flight were recorded in these experiments. Depending on the heat flux and subcooling level, bubbles staying on a heater were observed to eject liquid, vapor-liquid or fog-like jets flowing with velocities ~0.5–15 cm/s into the bulk liquid.^35–44^ In ground experiments, the jets formed in the direction opposite to the gravity vector whereas in low gravity they formed in both directions from the wire.^44^ These jets were concluded to be one of the important factors for overall heat transfer in subcooled boiling and the thermoscapillary force due to an interfacial temperature gradient (the Marangoni effect) was suggested as the dominant mechanism of jet formation.^35–44^ In contrast, a liquid plume rising from the bubble top was observed only in our ground experiments (Fig. 5) and its contribution to the heat flux appeared to be insignificant (Fig. 4(a).

## Discussion

Presented results demonstrate that energy was transferred away from the heater by evaporating water at the bubble base, condensing vapor on the bubble cap, and then by circulating flow to the bulk water. To evaluate basic parameters needed for understanding physical mechanisms underlying the energy transfer, we use data in Figs. 3 and 4 at *T*_h_ = 155°C, *q*_h_ = 0.6 MW/m^2^ taken as characteristic and properties of water and air^47–51^ (listed in SI1) at the saturation temperature of water *T*_sat_ = 100 °C at 1 atm, also as characteristic.

### Flows of vapor and air inside the bubble

The mass flow rate of liquid evaporating inside the bubble can be estimated as ṁ_v_ = q_h_S_h_/h_fg_~ 1.2 mg/s since the contribution of energy to heat the liquid flowing into the bubble and cool the condensate formed on the bubble cap is relatively small c_pl_(T_sat_−T_r_)/h_fg_~ 0.14, where c_pl_ and h_fg_ are the water specific heat capacity and latent-heat of vaporization and the room temperature T_r_ ≈ 25 °C. A liquid flows into the bubble over the heated surface at the bubble footprint in the layer whose thickness δ_1_ (Fig. 6a) can be evaluated from the mass balance δ_1_~ṁ_v_/πDρ_1_v_1_~ 0.2 mm by taking D~ 2 mm for the bubble base diameter and v_1_~ 1 mm/s for the liquid velocity according to data in Fig. 5. The time it takes to heat this liquid is ${\mathrm{t}}_{\mathrm{l}}{\mathrm{\sim \delta }}_{\mathrm{l}}^2{\mathrm{/\alpha }}_{\mathrm{l}}\sim 0.2{\mathrm{s}}$, α_1_ is the water thermal diffusivity. When the temperature of this liquid rises above T_sat_, tiny vapor bubbles would form and grow with a velocity of the order of several of m/s since the Jacob number Ja_h_ = ρ_1_c_pl_(T_h_−T_sat_)/ρ_v_h_fg_ ~ 164 corresponds to the inertia dominated regime.^52–55^ Once reaching the liquid surface, they burst and release vapor into the bubble interior. The length of the thin liquid-vapor region at the bubble footprint (1 in Fig. 6a) through which heat is transferred away from the heater can be estimated as l_1_~v_1_t_1_~ 0.24mm. To evaluate variation of the local temperature across the heater, we took the solution of a steady-state conduction problem for the temperature of a rectangular heater from which heat is conducted outside through its edges.^56^ In this case, the maximum and minimum local temperatures are respectively achieved at the heater center and the edges. Calculations presented in SI1 indicate that a difference between these values and the average heater temperature T_h_ increased from about 0.35 °C to 4.5 °C with raising T_h_ from 100 °C to 270 °C in our experiments (Fig. 4(a).

Estimates for the mass flux and velocity of the vapor formed in a thin layer at the bubble footprint (1 in Fig. 6a) yield j_v_~ṁ_v_/s_1_~0.6 mg/mm^2^·s and v_v_~j_v_/ρ_v_~1 m/s, where ${\mathrm{S}}_{\mathrm{l}}\sim {\mathrm{\pi D}}\sqrt {{\mathrm{\delta }}_{\mathrm{l}}^2 + {\mathrm{l}}_{\mathrm{l}}^2}$ ~ 2 mm^2^ is the area where liquid vaporizes. An estimate for the velocity of condensate formed on the bubble cap of area S~ 10 mm^2^ (data in Fig. 3d) v_lc_~ṁ_v_/ρ_1_S~ 0.1 mm/s shows that vapor condensation does not drive the liquid circulation around the bubble as v_lc_ is substantially smaller than flow velocities in Fig. 5.

Non-condensable air constituents (oxygen and nitrogen) brought by the liquid into the bubble are carried with the streaming vapor toward the bubble cap and remain there while the vapor condenses. The diffusive flux of the air away from the bubble surface can balance the vapor flow only within a thin layer of thickness ~d_a_/v_v_~ 40 μm, d_a_ is the diffusion coefficient of air constituents in water vapor (SI1). As the volume of this layer ~d_a_S/v_v_ is much smaller than the air volume in the bubble (data in Fig. 3e, c), air carried with the streaming vapor would accumulates away from the bubble cap (region 3 in Fig. 6a). Driven by the vapor flowing along the bubble cap (region 2 in Fig. 6a), the velocity with which the air circulates inside the bubble is v_a_~v_v_~ 1 m/s. Flow of vapor and air is laminar as the Reynolds numbers are ${\mathrm{Re}}_{\mathrm{v}}\sim \varrho _{\mathrm{v}}{\mathrm{v}}_{\mathrm{v}}{\mathrm{h}}/{\mathrm{\eta }}_{\mathrm{v}}$ ~ 120 and ${\mathrm{Re}}_{\mathrm{a}}\sim \varrho _{\mathrm{a}}{\mathrm{v}}_{\mathrm{a}}{\mathrm{h}}/{\mathrm{\eta }}_{\mathrm{a}}$ ~ 120, where h~ 3.6 mm is the bubble height (data in Fig. 3b) and $\varrho _{\mathrm{v}}$, η_v_ and $\varrho _{\mathrm{a}}$, η_a_ are the vapor and air density and dynamic viscosity. An estimate for the vapor volume l_1_S~ 2.4 mm^3^ is consistent with data on a change in the bubble volume ΔV in Fig. 3f caused by vapor condensation. The time to replenish the vapor condensed as the heating is turned off ~ρ_v_l_1_S/ṁ_v_~ 1.2s is also consistent with observed changes of the bubble size in heating cycles in flight and on Earth (data in Fig. 2a, c).

Once the three-phase contact line along the liquid layer inside a bubble resides on the well-wetted heater surface, the vapor region would be located above the heater surface as the interior contact angle is smaller than 90° (Fig. 6a). If the bubble grows by moving the liquid layer beyond the heater edge, vapor would come in contact with the PDMS surface since the interior contact angle of water on the hydrophobic PDMS surface is larger than 90°. This situation can occur only when the PDMS temperature is greater than the dew point of water vapor. Otherwise, the bubble would shrink due to vapor condensation. This negative feedback facilitates anchoring of a bubble to the heater if the temperature of the surrounding surface is below the dew point.

### Flow of liquid around the bubble

It is laminar as the Reynolds number is ${\mathrm{Re}}_{\mathrm{l}}\sim \varrho _{\mathrm{l}}{\mathrm{v}}_{\mathrm{l}}{\mathrm{h}}/{\mathrm{\eta }}_{\mathrm{l}}$~ 12, where the liquid velocity v_1_~ 1 mm/s (data in Fig. 5) and η_1_ is the liquid dynamic viscosity. This flow might be generated by the surface tension force along the bubble cap^3,8,14–17,57^ due to the temperature variation ΔT_b_ or by the shear stress exerted on the liquid-bubble interface by the flow of vapor and air inside the bubble. It is conceivable that both factors operate during the heating period, whereas the flow of vapor and air ceases when the heating is switched off (data in Fig. 5d, e). The stress balance at the bubble surface for the former η_1_v_1_/h~(dγ_1_/dT)ΔT_b_/h yields ΔT_b_~ 1.5×10^−3^ °C≪T_h_−T_r_. For the latter η_1_v_1_/h~η_v_v_v,τ_/h and/or η_1_v_1_/h~η_a_v_a,τ_/h, it yields 1−2 cm/s for the velocity of vapor v_v,τ_ and air v_a,τ_ tangent to the liquid-bubble interface that is much smaller that the velocity of vapor and air inside the bubble ~ 1 m/s.accomo These estimates indicate that the liquid flow around the bubble is highly sensitive to subtle processes at the bubble cap and requires a more careful study.

### Heat transfer at the bubble cap

To estimate temperatures of the liquid-vapor region T_e_ and at the bubble cap T_c_ (Fig. 6a), we used the Hertz-Knudsen equation for the vapor mass flux j_v_ with the accommodation coefficient ξ~ 0.01−0.1 for stagnant water surfaces.^58^ Calculations presented in SI1 yield T_e_≈114 °C, T_c_≈96 °C for ξ~ 0.01 and T_e_≈101 °C, T_c_≈99.7 °C for ξ~ 0.1. As the bubble cap temperature for both values of ξ is close to T_sat_ and T_s_ represents the bulk liquid temperature, the Nusselt number for heat transfer from the bubble cap to the surrounding liquid on Earth can be evaluated as

3 $${\mathrm{Nu}} = {\mathrm{q}}_{\mathrm{h}}{\mathrm{S}}_{\mathrm{h}}{\mathrm{h}}/{\mathrm{k}}_{\mathrm{l}}{\mathrm{S}}\left( {{\mathrm{T}}_{{\mathrm{sat}}}{\mathrm{ - T}}_{\mathrm{s}}} \right)$$

where k_1_ is the liquid thermal conductivity and variations of the heat flux at the bubble cap q_h_S_h_/S and the bubble height h with T_s_ were computed using data on q_h_, S and h at a fixed applied voltage in Fig. 3. Remarkably, values of Nu plotted in Fig. 6b for both heating regimes lie within a relatively narrow band, increasing with T_s_ from about 20 at room temperature to 140 at T_s_~ 80 °C. We compared these values with the data predicted by correlations for convective heat transfer from a vapor bubble of diameter condensing in a flow of subcooled water^59,60^ and the buoyancy convection heat transfer from the cap of a solid hemisphere.^61^ The former yielded Nu~ 2.5 and the latter Nu~ 11 for our experimental conditions (see SI1). These estimates clearly demonstrate that another mechanism contributes to energy transfer from the bubble cap to the surrounding liquid.

We attribute large values of Nu in Fig. 6b to periodic injection of a vapor-air mixture from the bubble caused by periodic overheating of the liquid layer near the bubble cap. Taking j_c_h_fg_ with j_c_~−S_1_j_v_/S for the heat flux of vapor condensing on the bubble cap, the time period and length it takes to heat the cool liquid flowing with the velocity v_1_ along the bubble cap from T_r_ to T_c_ can be estimated using the energy balance equation as ${\mathrm{t}}_{\mathrm{c}}\sim {\mathrm{\pi Ja}}_{\mathrm{c}}^2{\mathrm{\alpha }}_{\mathrm{l}}/4{\mathrm{v}}_{{\mathrm{vc}}}^2$ ~ 0.15 s and v_1_t_c_~ 0.15 mm, where Ja_c_ = ρ_1_c_pl_(T_c_−T_r_)/ρ_v_h_fg_~ 210 and v_vc_~j_c_/ρ_v_~ 0.2 m/s. When the local liquid temperature rises above T_c_, the rate of vapor condensation decreases and the local gas pressure under the bubble cap builds up, eventually jetting vapor mixed with air into the surrounding liquid. Once the layer of overheated liquid is blown away from the bubble cap and replaced by a colder liquid, the liquid temperature at this spot drops down below T_c_ causing the rate of vapor condensation to increase and thereby to stop gas injection. Then, the local liquid temperature begins to rise and the process repeats itself again and again. The estimate for the frequency of local overheating ~ 1/t_c_~ 7Hz, correlates well with the frequency range of bubble cap oscillations observed in flight and ground experiments. In low gravity, periodic injection of a vapor-air mixture created circulating tiny bubbles (Fig. 2a and SI2, video1) whose stability in the bulk liquid was caused by the residual air. On Earth, it generated plumes rising through the bulk liquid and releasing the trapped air by bubbling at the water surface (SI3, video2). In both cases, the rate of heat transfer from the bubble was drastically enhanced by increasing the area of the vapor-liquid interface. The proposed mechanism is consistent with the observation that Nu given by Eq. (3) (Fig. 6b) depends only on the water temperature. The flow circulation time within the cuvette t_f_~ 20 s (Fig. 5) is sufficient for the liquid flowing away from the bubble to transfer the acquired heat to the bulk by thermal conduction across closed streamlines as the characteristic length $\sqrt {2{\mathrm{\alpha }}_{\mathrm{l}}{\mathrm{t}}_{\mathrm{f}}}$~ 3 mm is comparable to half the cuvette width. Note that the frequency range of local liquid overheating is lower by more than an order of magnitude compared to intense capillary waves causing the violent emission of micrometer-sized bubbles from a condensing vapor bubble in the so-called microbubble emission boiling (MEB) regime.^55,62–64^

### Bubble growth and detachment

Bubble evolution is mainly governed by normal stresses at the bubble interface since the contribution of tangential stresses estimated above is relatively small. The normal stresses include the surface tension pressure, liquid hydrostatic pressure, and dynamic pressures of liquid and condensing vapor. The role of the dynamic pressure of liquid flow driven by the bubble growing at the timescale of the order of minutes (Fig. 3a–d) is insignificant as the ratio between its value and the surface tension pressure 4γ_1_/D is ~ 2×10^−6^. The contribution of the vapor dynamic pressure can be neglected^65–68^ only if the ratio ${\mathrm{\rho }}_{\mathrm{v}}{\dot{\mathrm V}}_{\mathrm{v}}^2/{\mathrm{\pi }}^2{\mathrm{D}}^3{\mathrm{\gamma }}_{\mathrm{l}}$ is smaller than 2.5×10^−6^, where *V*_v_ = qS_h_/ρ_v_h_fg_ is the vapor flow rate inside the bubble. An extremely low flow rate is required because the vapor velocity increases rapidly when the bubble neck shrinks radially. In our experiments at T_h_>100 °C where q_h_ was varying from 0.3 to 1.2 MW/m^2^ (Fig. 4a), this ratio ranging from 1.3×10^−4^ to 2.1×10^−3^ exceeded significantly this limitation. Nevertheless, the quasi-static equation for a pinned bubble^65–68^ that considers only the surface tension and hydrostatic pressures provides some understanding of the bubble behavior. In particular, the dimensionless solution of this equation predicts that the bubble shape is fully characterized by its relative height 2h/D and the Bond number Bo = (D/2L_c_)^2^. In boiling, the bubble height is determined by the thermal balance across the bubble cap that is expressed in terms of Nu specified by T_s_ (Fig. 6b). This feature explains why dimensionless characteristics of bubbles in ground experiments plotted against T_s_ for various values of q but all with Bo~ 0.16 for 100 °C would group together (Fig. 4c, d). It also explains a more elongated shape of bubbles observed in ground experiments compared to a spherical shape of bubbles formed in flight at Bo~ 1.6×10^−3^ (Fig. 4e). Within the framework of the quasi-static equation, the greatest value of the relative bubble height 2h_max_/D at a particular value of Bo for which the neck becomes zero is assumed to represent a bubble that divides into two parts in the neck region, one forming a departing bubble and the other a new pinned bubble.^66–68^ Expressions^67,68^ for the bubble at zero neck predict height h_max_ = 5.4 mm with the width 3.1 mm and volume V_d_ = 26.4 mm for Bo~ 0.16 and D~ 2 mm occurred in our ground experiments. While we observed a similar mode of bubble departure, a bubble at this instant was more elongated and its volume was two-three times smaller, depending on the heating regime (Fig. 3c). These estimates and experimental data on the bubble departure time (Fig. 4f, Eq. 2) indicate that the bubble pinch-off was strongly influenced by the vapor flow rate at the bubble base that is determined by the heat flux.

In conclusion, single-bubble boiling of water on a millimeter-sized well-wetted heater was recorded under normal gravity and low gravity in parabolic flights. The gravity contribution to the heat transfer was insignificant. The lifetime of a bubble on the heater varied from 1 to about 40 min, depending on the heating mode and water temperature. Due to self-adjustment of the bubble size, the heat flux provided by boiling rose linearly up to about 1.2 MW/m^2^ with increasing heater temperature and was not affected by a gradually rising water temperature. The application of high-voltage pulses increased the heat flux by about 10% at the same heater temperature. Estimates of basic flow and heat transfer parameters provided insight into physical mechanisms underlying single-bubble boiling. As the rate of heat transfer from the bubble cap exceeded predictions of convective models by more than an order of magnitude, it was attributed to periodic injection of a vapor-air mixture from the bubble to the surrounding liquid. While the quasi-static model for the bubble growth explained similarity in dependence of the bubble shape on water temperature for different heating regimes, the observed bubble pinch-off was strongly influenced by the vapor flow rate at the bubble base.

The fast response and stable operation of single-bubble boiling over a broad range of temperatures pave the way for development of new devices to control heat transfer by forming surface domains with distinct thermal properties and wettability. The bubble lifetime can be adjusted by changing the water temperature. The ability of heating water on millimeter scales far above 100 °C without an autoclave or a powerful laser provides a new approach for processing of biomaterials and chemical reactions. The replacement of PDMS with high-temperature insulation would make it possible to increase the heater temperature to 320–350 °C needed to initiate the spinodal decomposition of water.

## Methods

Flight and ground experiments were conducted in a rectangular UV-Vis quartz cuvette, Cole-Parmer, Chicago, IL (Fig. 1) equipped with a thin-film (1.3 mm thick) temperature resistance sensor (Innovative Sensor Technology, Las Vegas, NV). A PDMS slab (~ 6.3 mm thick) mounted at the cuvette bottom was fabricated from Sylgard 184, Dow Corning, Midland, MI. Heating DC voltage was provided with DIGI-35A and DIGI-185 (Electro Industries, Chicago, IL) and Hewlett Packard 6207B /Agilent 6207B. The voltage drop in electric circuits (Fig. 1) was recorded at 500 Hz with the data acquisition system U6-Pro Data Logger, LabJack, Lakewood, CO. The bubble motion was recorded at 13.95 fps with a DCC1545M-USB 2.0 CMOS camera, Thorlabs, Newton, NJ. The temperature below the water surface was recorded in ground experiments with a data logger TW-23039-64, Type K, Cole-Parmer, IL. HV pulses were generated with an amplifier Model 10/40 (slew rate 0.75 kV/μs) Trek, Lockport, NY and an Agilent Waveform Generator Model 33220a. The conditions of 31 experiments (3 in flights and 28 on Earth), the number of experiments replicated for the same heating regime, mean and standard deviations for the heat flux, heater temperature and bubble departure time, and the description and results of common statistical tests used for analysis of measurements to demonstrate the reproducibility of data are listed in SI1, Section 2. All measurements were included in the analysis.

## Electronic supplementary material

Flight setup, statistical analysis of experimental data, materials properties, and thermal characteristics

Flight experiments, SI2video1

Earth experiments, SI3video2

## Data Availability

The datasets generated during the current study are available from the corresponding author on reasonable request.

## Electronic supplementary material

**Supplementary information** accompanies the paper on the *npj Microgravity* website (10.1038/s41526-018-0055-y).
