# Supplementary material for: Single-bubble water boiling on small heater under Earth’s and low gravity
Source: NPJ Microgravity. 2018 Nov 2;4:21. doi: 10.1038/s41526-018-0055-y (PMC6214906; doi:10.1038/s41526-018-0055-y)
Supplement: Supplementary file 1 — Flight setup, statistical analysis of experimental data, materials properties, and thermal characteristics [file 41526_2018_55_MOESM1_ESM.pdf]

Flight setup, statistical analysis of experimental data, materials properties, and thermal characteristics

1. Flight setup

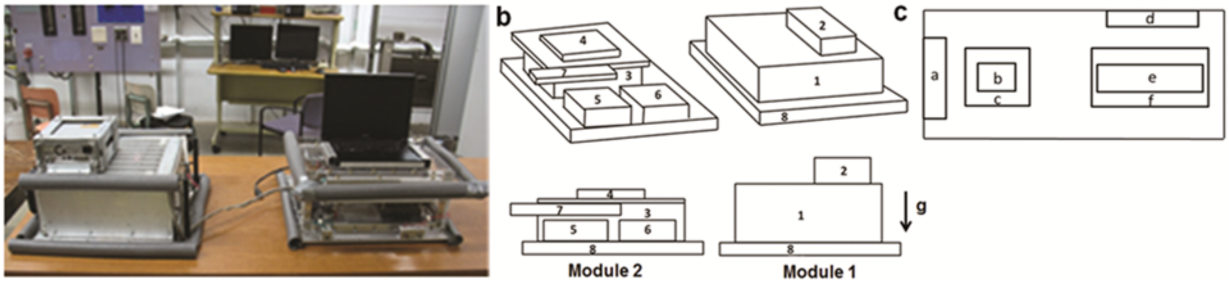

Figure S1. Flight setup: (a) Module 1 (*left*) and Module 2 with a laptop on the top (*right*), (b) Layout of components of Modules 1 and 2, (c) Layout of components within the enclosure in Module 2.

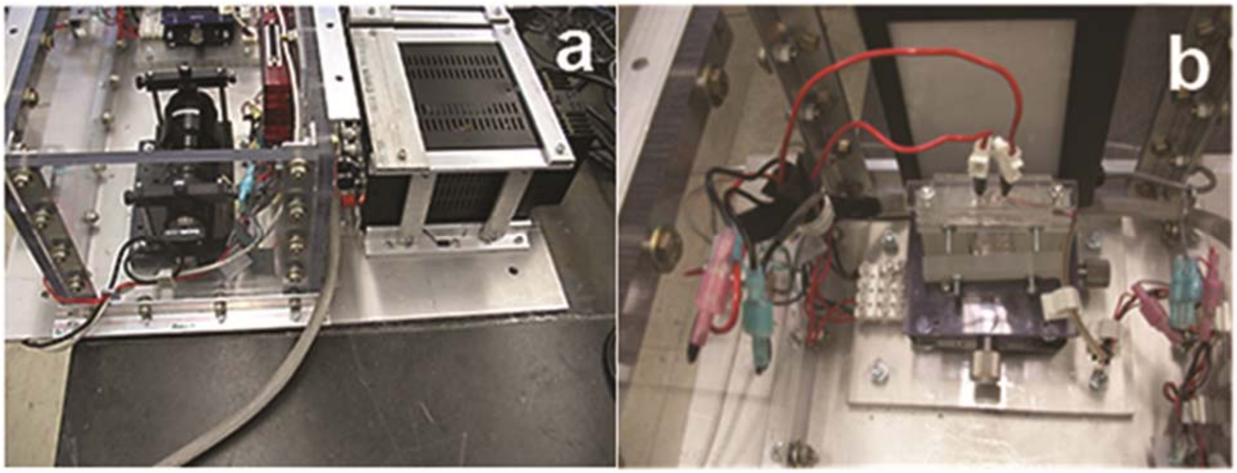

Figure S2. Module 2 components: (a) The camera mounted in the enclosure box (*left*) and the DC power source adjacent to the box (*right*), (b) Two rectangular cuvettes mounted on the X-Y positioning stage with the light source behind. The cuvette lid is equipped with a long electrode coated with Teflon that is connected to the HV amplifier.

The flight setup (Fig. S1(a)) consists of Module 1 and Module 2. Both modules are assembled on 1/8 in. aluminum 6061 T6 plate, 24 in. x 24 in., plates (8 in Fig. S1(b)) whose sharp edges box are padded with soft materials. In flight, these plates were bolted to the aircraft floor grid with AN-6 3/8 in. diameter steel bolts. Modules 1 and 2 were designed to withstand "g-forces" up to $9g_E$ along horizontal axis, up to $2g_E$ along positive vertical axis and up to $6g_E$ along negative vertical axis (crash conditions). Stress–strain analysis of the setup components was conducted using guidelines (references listed in the paper).[19]

Module 1 (Fig. S1(b)) comprises (1) a high voltage (HV) amplifier (Model 10/40, slew rate 0.75kV/μs, Trek, Lockport, NY) and (2) a function generator (Agilent Waveform Generator, Model 33220a). The HV amplifier and the function generator are secured with 2 in. x 2 in. x ¼ in. 90 degree aluminum 6061-T6 angle bars in the front, back and sides. These angle bars are fastened to the aluminum plate (8 in Fig. S1(b)) with 10-32 x 1/2 in. alloy steel screws. Module 2 (Fig. S1(b)) comprises (3) an enclosure box; (4) a laptop (Latitude E6500, Dell, Austin, TX) mounted on the top of the box; two low voltage direct current (DC) power sources (5, DIGI-35A, 0-30V at

0-3A; 6, DIGI-185, 0-18V at 0-5A; both from Electro Industries, Chicago, IL) and (7) a power strip with a 14AWG extension cord and a 15A circuit breaker. The top and sides of the enclosure box, 22 in. x 10.25 in. x 9.5 in., are constructed by bolting polycarbonate sheets to 1 in. x 1 in. x 1/8 in. 90 degree aluminum 6061-T6 angle bars with ¼-20 x 1 in. grade 8 alloy steel bolts. The enclosure top is extended to hold the power strip (7 in Fig. S1(b)) that distributes power to the HV amplifier, function generator, DC power sources, laptop, and lighting source. The camera is plugged into the laptop. The box angle bars, DC power sources, and components mounted inside the box (Figs. S1(c) and S2(a)) are fastened to 1/8 in. aluminum 6061 T6 plate (8 in Fig. S1(b)) with 10-32 x 1/2 in. alloy steel screws. The box sharp edges are padded with soft materials (Fig. S1(a)). Figures S1(c) and S2(a,b) show the layout of components within the enclosure box: (a) a light source (83-873 LED Backlight Illumination, Edmund Optics, Barrington, NJ); (b) two rectangular cuvettes (12.5mm x 12.5mm x 45mm, inner cross-section 10mm x 10mm; UV-Vis, Quartz Cuvettes, Cole-Parmer, Chicago, IL) mounted on (c) an X-Y positioning stage (39-930, 70mm Travel, X-Y Axis Positioning Stage, Edmund Optics, Barrington, NJ); (d) a data acquisition system (U6-Pro Data Logger, LabJack, Lakewood, CO); (e) a camera with lenses (DCC1545M-USB 2.0 CMOS Camera (1280 x 1024 pixels, monochrome, frame rates up to 250 fps, equipped with zoom lens MVL 7000, Thorlabs, Newton, NJ); signal in experiments was mainly recorded at 13.95 fps by the laptop (4 in Fig. S1) and (f) a camera/lens mount (03-665, V-Block Base with Clamps, Edmund Optics, Barrington, NJ).

Every cuvette in Module 2 is equipped with a thin-film temperature resistance sensor (P0K1.232.4W.B.010, Innovative Sensor Technology, Las Vegas, NV) acting as a resistance heater. The sensor is embedded into a polydimethylsiloxane (PDMS) slab (~6.3mm thick) mounted at the cuvette bottom such that the open sensor cover is lying in the horizontal plane of the slab surface (Fig. 1 in the paper). The slab was fabricated from Sylgard 184 using Silicone Elastomer Kit (Dow Corning, Midland, MI) and cured at room temperature. The sensor is connected in series with a DC power source to provide voltage for heating and a resistor (RadioShack, the nominal value of 8 Ohm/20W connected to DIGI-35A and 8 Ohm/10W connected to DIGI-185, the measured resistance of both, 8.3 Ohm, used in calculations). The voltage drop across this resistor is recorded at 500 Hz by the data acquisition system and used to compute the electrical current through the sensor, the power supplied to the sensor, and the sensor resistance. The sensor resistance is used to calculate its temperature from the linear calibration curve, resistance vs. temperature, provided by the manufacturer and validated by our measurements. The following modifications can be made in Earth's experiments: equip a cuvette with a temperature data logger (TW-23039-64, Type K, Cole-Parmer, IL) connected to the laptop to record the temperature slightly below the liquid surface, use another heating circuit to measure the sensor temperature (once heating is turned off) by applying 3V DC, and take a Hewlett Packard 6207B /Agilent 6207B DC power source, 0-160V at 0-0.2A to provide a larger DC voltage for heating liquid.

The cuvette is loaded with a test liquid and then closed with a plastic lid (Fig. S2(b)). A long energized electrode coated with Teflon is mounted in the cuvette lid (Fig. S2(b)) such that its tip is located in the vicinity of the heater. This electrode is connected to the HV amplifier to generate a strong alternating current (AC) electric field in the liquid. The voltage reversals in an AC field reduces the accumulation of electric charge in the liquid. Compared to conventional electric techniques using a DC field generated by inserting bare electrodes in the liquid, the chances of short circuit, sparking, electrochemical deterioration of a liquid and electro-corrosion of the system components would be greatly reduced. The electrical impedance between this electrode

and the sensor measured in distilled water by applying 200V AC decreases with the applied frequency from about several 10^4MOhm at 1 Hz to about several 10^2MOhm at 100 Hz and to several 10MOhm at 1kHz with the resistance capacitance phase shift of about 5^0 within this frequency range. A train of successive rectangular high-voltage (HV) pulses of alternating polarity, 3-4 kV at frequency up to 100 Hz, was applied to this electrode to affect boiling. Due to the high value of the electrical impedance, the power supplied to the water by these HV pulses was less than 0.2 W.

2. Experimental conditions and statistical analysis

The conditions of experiments carried out in parabolic flights and on Earth are listed in Tables S1-S3. Every flight brought a collection of data on the heat flux and heater temperature for the same heating regime through measurements for consecutive parabolic arcs. Values of the mean, standard deviation (STD) and relative standard deviation (%RSD) for these datasets are listed in Table S1. The Ljung-Box (LB) Q-test and the Anderson-Darling (AD) test in MATLAB 2017a (also see NIST/SEMATECH e-Handbook of Statistical Methods, http://www.itl.nist.gov/div898/handbook) were taken to explore whether deviations from the mean over the entire dataset for a particular heating regime were random quantities. The LB test was used to examine the presence of m (=20) autocorrelations of the residuals to assess the hypothesis that they are independently distributed, so that any observed correlations in the residuals come from randomness of the sampling process. This hypothesis is rejected if a p −value computed for the level of significance α that corresponds is relatively small. The AD test was used to assess whether residuals are from a population with a normal distribution. This hypothesis is rejected if a computed $p-$ value is relatively small. The p −values listed in Table S1 for LB and AD tests indicate that two hypotheses that the residuals were random quantities and that they were normally distributed cannot be rejected at a significance level of α=5%.

A similar approach was taken to analyze measurements in experiments on Earth with heating cycles when the heating DC voltage was periodically turned on for 20s and then off for 60s (Table S2). Every experiment included 30 cycles and was conducted twice. As a result, 60 values of heat flux were collected for the same heating regime through measurements for consecutive cycles in two experiments. The LB and AD statistical tests in MATLAB 2017a were used to explore whether deviations from the mean over the entire dataset for a particular heating regime were random quantities and whether they were normally distributed. The p −values listed in Table S2 for LB and AD tests indicate that two hypotheses that the residuals were random quantities and that they were normally distributed cannot be rejected at a significance level of α=5%. However, the dataset for 15V passed the LB test but failed the AD test at this α-level, indicating that the deviations from the mean were random quantities, but not normally distributed. A pass in the AD test for the 15V data set was observed at 1% significance level. Values of the mean, standard deviation and relative standard deviation for measurements in experiments on Earth with continuous heating are listed in Table S3.

For all experiments in Tables S1-S3, the heat flux q_h rises linearly with increasing the heater temperature T_h as

$q_h(kW/m^2) = (4.63 \pm 0.15)(T_h - 19.28°C)$ with the coefficient of determination $r^2 = 0.970$. Deviations between values given by this equation and measurements are normally distributed random quantities at the 95% confidence level with the p −values 0.917 and 0.179 for the AD and LB tests, respectively.

Table S1. Flight experiments

Day	DC voltage	Pulses	Number of parabolas	Heat flux, Mean ± STD, MW/m^2 / Heater temperature, Mean ± STD, ^{0}C	LB test; p- value	AD test; p- value
1	20V	3kV/20 kHz	28	0.533±0.012 (%RSD 2.28)	0.618	0.662
				119.7±10.1 (%RSD 8.4)	0.643	0.562
2	22.4V	4kV/20 kHz	27	0.630±0.016 (%RSD 2.48)	0.697	0.274
				146.1±11.3 (%RSD 7.8)	0.701	0.331
3	22.4V	4kV/10 kHz	30	0.621±0.022 (%RSD 3.55)	0.167	0.730
				152.6±16.5 (%RSD 10.8)	0.172	0.134

Table S2. Experiments on Earth with 30 heating cycles; DC voltage on for 20s and off for 60s; two experiments conducted under the same conditions

No	DC voltage	Pulses	Heat flux, Mean ± STD, MW/m^2 / Heater temperature, Mean ± STD, ^{0}C	LB test; p- value	AD test; p- value
1	15V	No	0.309±0.001 (%RSD 0.38)	0.935	0.023
			106.6±1.4 (%RSD 1.4)	0.871	0.072
2	20V	No	0.512±0.012 (%RSD 2.34)	0.243	0.508
			139.7±2.5 (%RSD 1.8)	0.318	0.254
3	22.4V	No	0.619±0.010 (%RSD 1.76)	0.937	0.073
			154.4±2.3 (%RSD 1.5)	0.827	0.202
4	25V	No	0.724±0.013 (%RSD 1.80)	0.738	0.101
			183.8±3.0 (%RSD 1.6)	0.721	0.250
5	30V	No	0.974±0.017 (%RSD 1.78)	0.102	0.223
			228.6±2.6 (%RSD 1.1)	0.066	0.345
6	35V	No	1.185±0.018 (%RSD 1.52)	0.228	0.071
			281.3±3.2 (%RSD 1.1)	0.274	0.125
7	20V	4kV/20Hz	0.526±0.012 (%RSD 2.28)	0.933	0.161
			128.3±2.8 (%RSD 2.2)	0.357	0.119
8	22.4V	4kV/20Hz	0.632±0.018 (%RSD 2.85)	0.384	0.067
			153.3±2.8 (%RSD 1.8)	0.586	0.292

Table S3. Experiments on Earth with continuous heating; three experiments (with pulses) and four experiments (without pulses) conducted under the same conditions

No	DC voltage	Pulses	Heat flux, Mean ± STD, MW/m^2	Heater temperature, Mean ± STD, ^{o}C
1	5V	No	0.048±0.002 (%RSD 4.15)	47.1±6.3 (%RSD 13.3)
2	10V	No	0.158±0.007 (%RSD 4.15)	61.2±9.3 (%RSD 15.2)
3	15V	No	0.325±0.013 (%RSD 4.01)	102.6±7.2 (%RSD 7.0)
4	20V	No	0.539±0.025 (%RSD 4.71)	136.0±8.2 (%RSD 6.0)
5	22.4V	No	0.654±0.030 (%RSD 4.53)	152.7±7.5 (%RSD 4.9)
6	25V	No	0.800±0.052 (%RSD 6.50)	185.9±7.3 (%RSD 5.0)
7	27.5	No	0.880±0.016 (%RSD 1.77)	225.8±9.6 (%RSD 4.3)
8	30V	No	1.000±0.024 (%RSD 2.44)	255.7±12.9 (%RSD 5.0)
9	35V	No	1.197±0.023 (%RSD 1.92)	268.9±13.5 (%RSD 5.0)
10	5V	4kV/20Hz	0.071±0.003 (%RSD 4.53)	34.6±3.3 (%RSD 9.4)
11	10V	4kV/20Hz	0.184±0.005 (%RSD 2.74)	48.3±3.3 (%RSD 9.4)
12	15V	4kV/20Hz	0.331±0.010 (%RSD 2.92)	85.0±5.0 (%RSD 5.8)
13	20V	4kV/20Hz	0.543±0.012 (%RSD 2.21)	126.5±1.2 (%RSD 1.0)
14	22.4V	4kV/20Hz	0.681±0.010 (%RSD 1.46)	141.3±4.7 (%RSD 3.4)
15	25V	4kV/20Hz	0.793±0.007 (%RSD 0.86)	176.5±5.0 (%RSD 2.8)
16	30V	4kV/20Hz	1.040±0.020 (%RSD 1.92)	231.4±12.6 (%RSD 5.4)
17	20V	4kV/1Hz	0.491±0.007 (%RSD 1.43)	132.2±0.8 (%RSD 0.6)
18	20V	4kV/10Hz	0.501±0.011 (%RSD 2.19)	131.1±1.1 (%RSD 0.9)

19	20V	4kV/50Hz	0.517±0.008 (%RSD 1.55)	127.6±1.1 (%RSD 0.8)
20	20V	4kV/100Hz	0.495±0.012 (%RSD 2.42)	130.3±1.6 (%RSD 1.2)

Measurements of the departure time of the first bubble formed on the heater after applying DC voltage and the liquid temperature T_s at this instant for experiments on Earth are listed in Tables S4 and S5. For both heating modes, the departure of this bubble occurred in the range of liquid bulk temperatures T_s ~ 50-80℃ and showed the same dependence on the heat flux
$T_s(℃) = (27.2 \pm 0.93)q_h(MW/m^2) + 44.96 \pm 1.13℃$ with $r^2 = 0.966$.
Deviations between values given by this equation and measurements are normally distributed random quantities at the 95% confidence level with the p −values 0.588 and 0.564 for the AD and LB tests, respectively.

Table S4. Experiments on Earth; 30 heating cycles 20s on/60s off; two experiments conducted under the same conditions.

No	DC voltage	Pulses	Departure time of the first bubble, Mean ± STD, min	Liquid temperature T_s Mean ± STD, °C
1	20V	No	37.0±0.9 (%RSD 2.5)	58.5±3.5 (%RSD 6.0)
2	22.4V	No	33.7±1.9 (%RSD 5.6)	62.0±2.8 (%RSD 4.5)
3	25V	No	33.0±0.9 (%RSD 2.7)	64.0±1.4 (%RSD 2.2)
4	30V	No	25.7±1.9 (%RSD 7.4)	72.5±2.1 (%RSD 2.9)
5	35V	No	21.7±1.9 (%RSD 8.7)	79.5±2.1 (%RSD 2.6)
6	20V	4kV/20Hz	35.7±2.8 (%RSD 7.8)	59.5±2.1 (%RSD 3.5)
7	22.4V	4kV/20Hz	35.0±1.9 (%RSD 5.4)	62.5±0.7 (%RSD 1.1)
8	25V	4kV/20Hz	34.3±0.9 (%RSD 2.6)	66.5±2.1 (%RSD 3.2)
9	30V	4kV/20Hz	27.0±1.9 (%RSD 7.0)	74.0±2.8 (%RSD 3.8)
10	35V	4kV/20Hz	20.3±2.0 (%RSD 9.8)	80.0±1.4 (%RSD 1.8)

Table S5. Experiments on Earth with continuous heating; three experiments (with pulses) and four experiments (without pulses) conducted under the same conditions

No	DC voltage	Pulses	Departure time of the first bubble, Mean ± STD, min	Liquid temperature T_s Mean ± STD, °C
1	15V	No	9.1±0.5 (%RSD 5.5)	54.7±3.2 (%RSD 5.9)
2	20V	No	6.9±1.0 (%RSD 14.5)	61.7±1.5 (%RSD 2.4)
3	22.4V	No	5.7±1.2 (%RSD 21.0)	63.0±2.0 (%RSD 3.2)
4	25V	No	3.8±0.6 (%RSD 15.8)	64.3±2.5 (%RSD 3.9)
5	30V	No	2.6±0.5 (%RSD 19.2)	69.0±5.3 (%RSD 7.7)
6	35V	No	1.6±0.5 (%RSD 31.2)	77.0±2.0 (%RSD 5.0)
7	15V	4kV/20Hz	8.0±0.8 (%RSD 10.0)	54.3±3.5 (%RSD 6.4)
8	20V	4kV/20Hz	5.6±1.6 (%RSD 28.6)	59.3±3.1 (%RSD 5.2)
9	22.4V	4kV/20Hz	5.0±0.3 (%RSD 6.0)	62.3±1.5 (%RSD 2.4)
10	25V	4kV/20Hz	3.6±0.4 (%RSD 15.8)	65.7±2.1 (%RSD 3.2)
11	30V	4kV/20Hz	3.1±0.4 (%RSD 12.9)	71.0±3.6 (%RSD 5.1)
12	35V	4kV/20Hz	1.7±0.3 (%RSD 17.6)	79.0±1.0 (%RSD 1.3)

2. Materials properties

Table S6. PDMS and metals at room temperature

Material	Specific gravity, kg/m^3	Specific heat, kJ/kg·K	Thermal conductivity, W/m·K	Thermal diffusivity, m^2/s
PDMS*[1]	970	1.46	0.15	$1.06 \cdot 10^{-7}$

Silver*[2]	10490	0.23	406	$1.68\cdot10^{-4}$
Copper*[3]	8940	0.39	385	$1.10\cdot10^{-4}$
Platinum*[4]	21400	0.13	71.6	$2.57\cdot10^{-5}$

*[1]Mark, J. E. (ed). Polymer Data Handbook, 2nd Ed (Oxford Univ. Press, 2009)
*[2]https://www.engineeringtoolbox.com/metal-alloys-densities-d_50.html
*[3]https://www.engineeringtoolbox.com/specific-heat-metals-d_152.html
*[4]https://www.engineeringtoolbox.com/thermal-conductivity-metals-d_858.html

Table S7. Water[47-49] at 1 atm, T_{sat}=100^{0}C

Density, liquid	Density, vapor	Specific heat, liquid	Latent heat of vaporization	Surface tension	Temperature derivative of surface tension
958 kg/m^3	0.6 kg/m^3	4.2 kJ/kg·K	2,257 kJ/kg	58.9 mN/m	-0.19 mN/m·K

Viscosity, liquid	Thermal conductivity, liquid	Thermal diffusivity, liquid	Prandl, liquid	Viscosity, vapor	Liquid volumetric expansion
0.28 mPa·s	0.68 W/m·K	$1.7\cdot10^{-7}$ m^2/s	1.72	$1.2\cdot10^{-2}$ mPa·s	$7.5\cdot10^{-4}$ 1/K

The Antoine equation for saturated water vapor pressure [47-49]

$$\log_{10}P_{eq,w} = A - B/(C+T) \tag{S1}$$

with A=8.14019, B=1810.94, C=244.485, where the vapor pressure is in Torr and the temperature is in °C.

Table S8. Air[47-49] at 100^{0}C

Density	Viscosity
0.95 kg/m^3	$2.2\cdot10^{-2}$ mPa·s

Diffusion coefficients of molecules in water vapor were estimated using the empirical correlation proposed by Fuller, Schettler, and Giddings:[50,51]

$$d_{AB} = \frac{10^{-3}T^{1.75}(1/M_A+1/M_B)^{1/2}}{P\left[(\sum v)_A^{\frac{1}{3}}+(\sum v)_B^{\frac{1}{3}}\right]^2},$$

where d_{AB} is the diffusivity in cm^2/s, P is the total pressure in atm, M is the molecular weight, T is the temperature in K, and $\sum v$ is the diffusion volume of a component.[50,51]

Table S9. Diffusion coefficients in water vapor at 1 atm and 100^{0}C

Molecule	Molecular Weight	v	d_{AB}, cm^2/s
O_2	32	16.6	0.39
N_2	28	17.9	0.39
H_2O	18	12.7	0.48

3. Heat loss evaluation

When a heating voltage is applied to the heater (Fig. 1 in the paper), transient heat conduction from the heater into the PDMS slab lasts $\sim b^2/\alpha_s \sim 340s$, where b is the slab thickness and α_s is the PDMS thermal diffusivity (Table S6). During this time, the rate of heat transfer into the slab can be estimated by taking the expression[20] for the heat flux at the surface of a semi-

infinite solid whose surface temperature changes instantaneously from the room temperature $T_r \approx 25°C$ to T_h

$$\dot{Q}_{trans}(t) \approx k_s S_h (T_h - T_r)/\sqrt{\pi \alpha_s t} \quad \text{with} \quad k_s S_h/\sqrt{\pi \alpha_s t} < 6.9 \cdot 10^{-4}\,W/°C \quad \text{for } t > 3s \qquad (S2)$$

where S_h is the heater surface area and k_s is the PDMS thermal conductivity (Table S6). After that, the rate of heat transfer into the slab can be estimated by using the expression[20] for the heat flux from a circular disk on the surface of a semi-infinite solid with area equal to the heater surface area

$$\dot{Q}_{steady} \approx 4k_s (T_h - T_r)\sqrt{S_h/\pi} \qquad \text{with} \qquad 4k_s\sqrt{S_h/\pi} = 7.3 \cdot 10^{-4}\,W/°C \qquad (S3)$$

Transient heat conduction from the heater through the silver and the copper wires (Fig. 1 in the paper) lasts less than 1s due to a high thermal diffusivity of metals (Table S6). After that, the rate of heat transfer can be estimated by calculating the total thermal resistance R_{total} of the silver and copper wires connected with the soldering joints (Fig. 1 in the paper). The wire thermal resistance is equal to its length divided by the cross-sectional area and the thermal conductivity (Table S6).The thermal resistance of the soldering joint can be estimated by using the expression[20] for the conduction shape factor of a sphere of diameter $c \sim 1.1$ mm with temperature T_j buried in a semi-infinite solid with temperature T_r

$$\dot{Q}_{sphere} = 2k_s \pi c (T_j - T_r)/(1 - \frac{c}{4z}),$$

where $z \sim 3$mm is the distance from the sphere center to the solid surface. Taking the silver wire to be connected in series with the copper wire and the soldered joint that are connected in parallel, we obtain

$$\dot{Q}_{power\,lead} \approx (T_h - T_r)/R_{total} \qquad \text{with} \qquad 1/R_{total} \approx 2.2 \cdot 10^{-3}\,W/°C \qquad (S4)$$

The presented estimates indicate that heat loss from the heater occurred mainly through the power lead wires. The total heat loss given by Eq. (S3) and Eq. (S4) increased with raising the heater temperature T_h from about 9% at $T_h \approx 50°C$ to 13% at $T_h \approx 270°C$.

4. Thermal regime of water

The cuvette in Earth's experiments was equipped with a temperature data logger to record the temperature T_s slightly below the water surface. Relative changes of T_s with time t after several tens of seconds of continuous heating are well approximated by a linear expression with a single time scale: $\ln[(T_m - T_s)/(T_m - T_r)] = -t/\tau_h$, where T_m is the final steady-state temperature, $T_r \approx 25°C$ is the room temperature, and τ_h is the characteristic time scale. The values of T_m and τ_h were obtained by maximizing the square of the Pearson correlation coefficient r^2 between $\ln[(T_m - T_s)/(T_m - T_r)]$ and time t. Figure S3 illustrates the dependence of T_m and τ_h on the applied heating DC voltage. The corresponding values of r^2 lie in the range 0.962 ± 0.014.

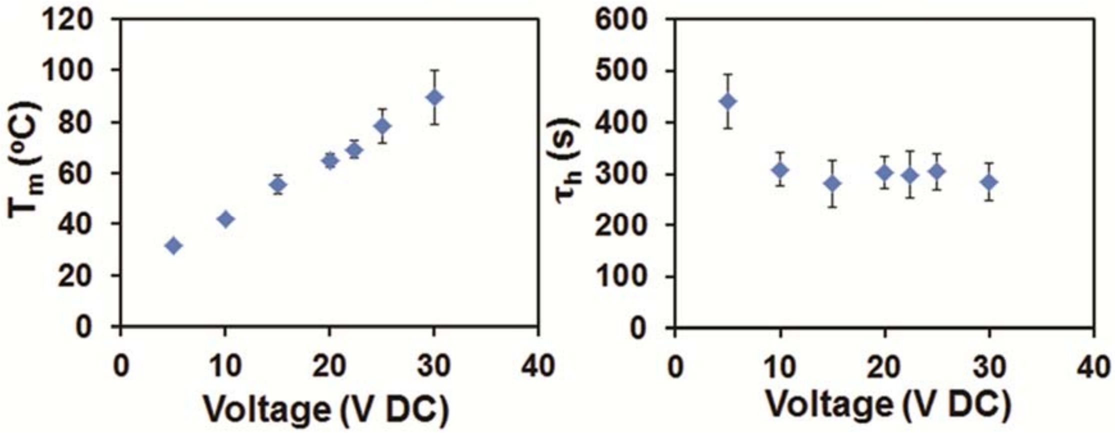

Figure S3. The dependence of T_m and τ_h on applied heating DC voltage.

5. Calculation of the bubble volume and cap surface area

Video images of the bubble evolution indicated that the bubble contour could be approximated as axisymmetric and thereby expressed as $r(z)$, where r is the distance from a point on the bubble contour to the central axis perpendicular to the heater plane and z is the distance from this point to the heater plane. Accordingly, the bubble volume V and the bubble cap surface area S are $V = \pi \int_0^h r^2 dz$ and $S = 2\pi \int_0^h r\sqrt{1 + (dr/dz)^2} dz$, where h is the bubble height.

To find $r(z)$, we formed a uniform grid of $n + 1$ points along the central axis of a bubble in a video image ($z_i = (i-1)\,h/n$ for $i = 1, \dots, n+1$) and measured distances from points on the bubble contour with each of these z-coordinates to the central bubble axis using NIS Advanced Imaging software (Nikon Instruments, Melville, NY). The integrals were then calculated following the trapezoidal rule:

$$V = \frac{\pi h}{2n} \sum_{i=1}^{n} (r_{i+1}^2 + r_i^2)$$

$$S = \frac{\pi h}{n} \sum_{i=1}^{n} \left(r_{i+1}\sqrt{1 + (dr/dz)_{i+1}^2} + r_i\sqrt{1 + (dr/dz)_i^2} \right),$$

where derivatives dr/dz at the internal and boundary points of the grid were computed as $\left(\frac{dr}{dz}\right)_i = \frac{r_{i+1} - r_{i-1}}{2h}$ at $1 < i < n$, $\left(\frac{dr}{dz}\right)_1 = \frac{r_2 - r_1}{h}$ and $\left(\frac{dr}{dz}\right)_{n+1} = \frac{r_{n+1} - r_n}{h}$.

It was found that taking 11 points along the bubble contour provided sufficient accuracy in estimating V and S. In particular, the errors in calculation of the volume and surface area of a spherical cap of diameter D and height h, $V_{cap} = \frac{\pi h}{6}(3D^2/4 + h^2)$ and $S_{cap} = \pi(D^2/4 + h^2)$, based on measurements of coordinates of 11 points are respectively less than 1% and 3%.

6. Calculations of temperature difference across the heater

The steady-state conduction problem for the temperature of a rectangular heater ($2L \times 2l$) of thickness H is used for estimating variations of the local temperature across the heater. The heat generation is considered to be uniform. The heat is transferred away from the heater only through its edges that are maintained at the temperature T_0. The solution if this two-dimensional problem is given by the following equation[56]

$$T - T_0 = \frac{\dot{Q}L}{4Hk_h l}\left\{\frac{1}{2}\left[1 - \left(\frac{x}{L}\right)^2\right] - \frac{16}{\pi^3}\sum_{n=0}^{\infty}\frac{(-1)^n}{(2n+1)^3}\left[\frac{\cosh\frac{(2n+1)\pi y}{2L}}{\cosh\frac{(2n+1)\pi l}{2L}}\right]\cos\frac{(2n+1)\pi x}{2L}\right\},$$

where is k_h is the heater thermal conductivity, $\dot{Q}$ is the total generated heat and $-L \le x \le L$, $-l \le y \le l$. The maximum temperature T_{max} is achieved at the heater center at $x = y = 0$ and the minimum temperature at its edges

$$T_{max} - T_0 = \frac{\dot{Q}L}{4Hk_h l}\xi_m\left(\frac{l}{L}\right) \text{ with } \xi_m\left(\frac{l}{L}\right) = \frac{1}{2} - \frac{16}{\pi^3}\sum_{n=0}^{\infty}\frac{(-1)^n}{(2n+1)^3\cosh\frac{(2n+1)\pi l}{2L}}$$

The average temperature T_{aver} of the heater is $T_{aver} - T_0 = \frac{\dot{Q}L}{4Hk_h l}\xi_a\left(\frac{l}{L}\right)$

$$\text{with } \xi_a\left(\frac{l}{L}\right) = \frac{1}{3} - \frac{64}{\pi^5}\sum_{n=0}^{\infty}\frac{\tanh\frac{(2n+1)\pi l}{2L}}{(2n+1)^5} = \frac{1}{3} + \frac{64}{\pi^5}\left[\sum_{n=0}^{\infty}\frac{1-\tanh\frac{(2n+1)\pi l}{2L}}{(2n+1)^5} - \varsigma(5)\left(1 - \frac{1}{2^5}\right)\right],$$

where the Riemann zeta function $\varsigma(5) = \sum_{n=1}^{\infty}\frac{1}{n^5} = 1.0369277551\ldots$,

Taking $2L = 2\text{mm}$ and $2l = 2.3\text{mm}$, we obtain $\xi_m = 0.2914$ and $\xi_a = 0.1393$ that yields

$$T_{max} - T_0 = 0.0728\frac{\dot{Q}L}{Hk_h l} \text{ and } T_{aver} - T_0 = 0.0348\frac{\dot{Q}L}{Hk_h l} \quad \text{(S5)}$$

In in our experiments, the average heater temperature increased from 100^0C to 270^0C with raising the total heat from about 0.5W to 5.5W (Fig. 4(a) in the paper). Taking $H = 0.65\text{mm}$ (Fig. 1(d) in the paper) and the platinum thermal conductivity (Table S6) for k_h in Eq. (S5), we find that $T_{max} - T_{aver}$ and $T_{aver} - T_0$ increased from about 0.35^0C to 4.5^0C with raising $\dot{Q}$ from 0.5W to 5.5W.

7. Heat transfer at the bubble cap

7.1. Temperatures in the liquid-vapor region and at the bubble cap

To estimate temperatures of the liquid-vapor region T_e and the bubble cap T_c (Fig.6(a) in the paper), we used the Hertz-Knudsen equation for the dependence of the vapor mass flux j_v on the vapor pressure P_v and temperature T

$$j_v = \frac{2\xi}{2-\xi}\sqrt{\frac{M_w}{2\pi R_{gas}T}}\left(P_{eq,w}(T) - P_v\right) \quad \text{(S6)}$$

with the water molecular weight M_w, the gas constant R_{gas}, the saturated vapor pressure $P_{eq,w}(T)$ computed using the Antoine equation Eq. (S1) and the accommodation coefficient $\xi \sim 0.01 - 0.1$ for stagnant water surfaces.[58] P_v inside the bubble can be approximated as 1atm as $dT/dP_{eq,w} \sim 2.8 \cdot 10^{-4}$K/Pa and thereby temperature variations caused by the vapor flow pressure $\rho_v v_v^2 \sim 0.6$Pa and surface tension pressure $4\gamma_l/D \sim 110$Pa are insignificant. The flux of vapor condensing at the bubble cap can be estimated as $j_c \sim -S_l j_v/S$ with $S = 10\text{m}^2$.

For our experimental conditions, Eq. (S6) yields $T_e \approx 114$°C, $T_c \approx 96$°C for $\xi \sim 0.01$ and $T_e \approx 101$°C, $T_c \approx 99.7$°C for $\xi \sim 0.1$.

7.2. Convective Nusselt numbers

It is instructive to compare the Nusselt numbers in Fig. 6(b) in the paper with the data predicted by correlations for convective heat transfer from a vapor bubble flowing in subcooled water[59] and the buoyancy convection heat transfer from the cap of a solid hemisphere.[61] The Nusselt number for the convective heat transfer from a vapor bubble of diameter $D(t)$ condensing in a flow of subcooled water is given by the following expression[59]

$$Nu(t) = 0.6Re^{1/2}Pr_l^{1/3}\left(1 - 1.20Ja^{9/10}Fo_0^{2/3}\right) \quad \text{(S7)}$$

where $Re = \varrho_l v_B D/\eta_l$ is the Reynolds for the bubble velocity v_B relative to the liquid, Pr_l is the liquid Prandtl number, $Ja = \rho_l c_{pl}\Delta T_{sub}/\rho_v h_{fg}$ is the Jacob number for the water subcooled

temperature ΔT_{sub}, and $Fo_0 = \alpha_l t/D_0^2$ with the initial bubble diameter D_0 accounts for thickening of the thermal boundary layer around a bubble with time t.

Experiments[59] were conducted over the range of Re and Ja close to our experiments. As the first term in Eq. (S7) represents the Nusselt number similar to that for a plate,[60] Eq. (S7) for our experimental conditions with $D_0 = h$, $v_B = v_l = 1\mathrm{mm/s}$ (Fig. 5 in the paper) and $\Delta T_{sub} = 75°\mathrm{C}$ yields Nu decreasing from the initial 2.5 to zero at $\sim 2.6\mathrm{s}$.

An attempt to use data on the buoyancy convection heat transfer from the cap of a solid hemisphere[61] of radius r_H by taking $r_H = h$ and the Rayleigh number $Ra_H = g\beta_l \Delta T r_H^3/\nu_l \alpha_l \sim 5 \cdot 10^5$, where β_l is the liquid volumetric expansion, predicted $v_H \sim \alpha_l Ra_H^{1/2}/r_H \sim 3.3\,\mathrm{cm/s}$ for the liquid velocity that overestimates velocity measurements in our Earth's experiments (Fig.5 in the paper) and yields

$$Nu_H = 0.415 Ra_H^{1/4} \sim 11$$
